# Costing RTS,S introduction in Burkina Faso, Ghana, Kenya, Senegal, Tanzania, and Uganda: A generalizable approach drawing on publicly available data

**DOI:** 10.1016/j.vaccine.2015.10.079

**Published:** 2015-11-27

**Authors:** Katya Galactionova, Melanie Bertram, Jeremy Lauer, Fabrizio Tediosi

**Affiliations:** a Department of Epidemiology and Public Health, Swiss Tropical and Public Health Institute, Basel, Switzerland; b University of Basel, Basel, Switzerland; c Health Systems Governance and Financing, World Health Organization, Switzerland

**Keywords:** Malaria, Costing, Vaccine introduction, Vaccine, RTS,S

## Abstract

Recent results from the phase 3 trial of RTS,S/AS01 malaria vaccine show that the vaccine induced partial protection against clinical malaria in infants and children; given the high burden of the disease it is currently considered for use in malaria endemic countries. To inform adoption decisions the paper proposes a generalizable methodology to estimate the cost of vaccine introduction using routinely collected and publicly available data from the cMYP, UNICEF, and WHO-CHOICE. Costing is carried out around a set of generic activities, assumptions, and inputs for delivery of immunization services adapted to a given country and deployment modality to capture among other factors the structure of the EPI program, distribution model, geography, and demographics particular to the setting. The methodology is applied to estimate the cost of RTS,S introduction in Burkina Faso, Ghana, Kenya, Senegal, Tanzania, and Uganda. At an assumed vaccine price of $5 per dose and given our assumptions on coverage and deployment strategy, we estimate total economic program costs for a 6–9 months cohort within $23.11–$28.28 per fully vaccinated child across the 6 countries. Net of procurement, costs at country level are substantial; for instance in Tanzania these could add as much as $4.2 million per year or an additional $2.4 per infant depending on the level of spare capacity in the system. Differences in cost of vaccine introduction across countries are primarily driven by differences in cost of labour. Overall estimates generated with the methodology result in costs within the ranges reported for other new vaccines introduced in SSA and capture multiple sources of heterogeneity in costs across countries. Further validation with data from field trials will support use of the methodology while also serving as a validation for cMYP and WHO-CHOICE as resources for costing health interventions in the region.

## 1 Introduction

RTS,S vaccine against *Plasmodium falciparum* malaria has demonstrated moderate levels of efficacy in phase 3 trials in Africa and is currently considered for use within the Expanded Programme on Immunization (EPI) in endemic countries [1]. Tested in children (5–17 months) and young infants (6–12 weeks) the vaccine has shown high initial efficacy, but its protection waned quickly with efficacy against clinical disease at 36.3% and 25.9% depending on age at immunization [2], [3]. The vaccine is thus evaluated as an additional tool for preventing clinical disease in children, not a replacement for existing malaria preventive, diagnostic, and treatment measures [4]. Despite being partially effective, modelling studies predict RTS,S to have a substantial public health impact on disease burden [5].

With a positive scientific opinion on vaccine efficacy and safety issued by the European Medicines Agency earlier this year, the WHO is expected to follow-up with a policy recommendation on the use of RTS,S [4]. As countries, donors, and international organizations consider RTS,S introduction, data on program costs are needed. Combined with effectiveness, cost data allow policy makers to assess the value of this new intervention in the context of a malaria control strategy [6], [7]. Decision-making at the country level is further concerned with financing and feasibility of mobilizing and maintaining the level of resources to support the new vaccine [8], [9].

To inform these decisions the study proposes a generalized methodology to estimate costs of RTS,S introduction in the EPI program. We apply it to assess these costs in Burkina Faso, Ghana, Kenya, Senegal, Tanzania, and Uganda. Costing is implemented around a set of generic and easily modifiable assumptions describing vaccine introduction; these capture among other factors the structure of the EPI program, distribution model, geography, and demographics particular to the setting. Our findings illustrate the broad ranges for costs of introduction dependent on the level of spare capacity to accommodate the vaccine. We show that differences in EPI structure, cost of labour, and intensity of use of resources within the system translate into significant differentials in cost of vaccine delivery between settings.

## 2 Vaccine presentation and deployment strategy

We base our assumptions about vaccine presentation on [10], [11]. RTS,S is a monovalent lyophilized vaccine reconstituted with an adjuvant; both require cold chain storage at 2–8° C. The vaccine and the diluent are clipped together with a packed volume of 9.68 cm^3^ per two-vial package. Two doses per vial are yielded after reconstitution.

Clinical trial data suggest that at least three doses are required for protection against malaria. Following the trial design [11], we evaluate four immunization schedules: a program targeting infants at 6, 10 and 12 weeks of age (6–12 weeks); children at 6, 7.5 and 9 months (6–9 months); and a four dose schedule in the two age groups including vaccination at 18 months after the 3rd dose. We assume national roll-out to scale through routine outlets with immunization schedule tied to DTP. For 6–9 months implementation we assume first and third doses to be administered along with vitamin A and measles vaccine and treat these as routine immunization visits for purposes of costing. New, out-of-routine schedule, visits are assumed for the second dose in 6–9 months and fourth doses.

## 3 Methods

### 3.1 Perspective

A broad provider perspective is adopted in this evaluation; all resources required to introduce the RTS,S into the national program are included in the analysis.

### 3.2 Scope

We estimate both the economic and the financial costs of introducing the malaria vaccine into the EPI. Financial costs represent actual expenditures on goods and services. Economic costs “define costs in terms of the alternative uses that have been forgone by using a resource in a particular way”; these include, in addition to the financial costs, a valuation of resources that do not have financial transactions (i.e. donated goods and services or capital goods, health care resources diverted from other uses or shared with other health programs, and inputs whose prices are distorted [12]). Given paucity of data on the level of existing capacity in the system, the financial costs are estimated under an assumption of 100% spare capacity to accommodate the vaccine. In reality, however, some countries might need to invest in scaling-up across a range of service inputs be it cold chain, vehicle fleet, or labour to deliver the new intervention. Assumptions for capacity scale-up, while not used to produce estimates here, are shared in Supplementary File S1. The economic costs are implicitly evaluated under the assumption of no spare capacity. Taken together, the two sets of estimates give a wide but informative range for potential vaccine introduction costs.

### 3.3 Assumptions for delivery of immunization services

We defined a set of essential activities for RTS,S introduction based on WHO [7], [13], [14], USAID [15], [16], and other guidelines on immunization [17], [18]. Assumptions about operational aspects of the program were further informed by published micro-costing studies that evaluated the introduction of new vaccines in low-income countries [19], [20], [21], [22], [23]. No campaigns or additional outreach activities outside of the routine EPI delivery were considered. For deployment modalities requiring out-of-routine schedule visits we assumed lower coverage rates and adjusted service delivery assumptions to reflect the longer time needed to administer the vaccine, increased IEC, and supervision to maintain coverage. Assumptions on activities costed and key inputs are presented in detail in Table 1. Scenarios by schedule are highlighted in Supplementary Table S1.

### 3.4 Resource lists

Resource lists were populated following the activities defined for vaccine introduction. These were adapted to countries using comprehensive Multi-Year Plans (cMYP) for immunization [24], [25], [26], [27], [28], [29]. The latter were particularly useful in identifying staff categories, equipment, and quantities of resources used by the EPI program at each level. Resource lists and prices for each country are documented in Supplementary File S2.

### 3.5 Input prices and unit costs

Data on input prices and unit costs came from several sources. Information on wages by level of EPI staff, per-diems, and some other line items were taken from cMYP [24], [25], [26], [27], [28], [29]. We also used data from the UNICEF [30] for prices of immunization supplies, and related equipment. Additionally data from the WHO-CHOICE databases were used to cost facility rental, hotel rates, fuel, and other commodities [31]. Prices of commodities obtained from the international price lists were adjusted for freight, insurance and wastage [13].

Shared inputs were attributed to RTS,S based on the direct allocation [12]; except for vaccinators whose contribution to the new intervention was costed based on time required to administer the vaccine. Similarly, cost of cold chain and vehicles were allocated to RTS,S based on use; for these inputs use refers to the volume of the vaccine and immunization supplies and time for storage or distance over which these were transported or stored.

Cost of capital items including vehicles, facilities, and equipment was annualized over the respective estimated useful life. Expenditures associated with activities held in the introductory stage were considered capital goods and were annualized and discounted over 5 years at 3%.

### 3.6 Algorithm for calculating cost of immunization

For each activity outlined in Table 1 we defined formulas to combine price and unit cost data with assumptions on resource use. These start with the general representation of an activity in terms of cost components, break it down to micro inputs and detail how unit costs are combined with quantities to obtain total cost per activity. Formulas are presented in Supplementary File S3; these are generalized and could be adapted to alternate assumptions on service delivery. Costs are estimated for a single cohort of surviving infants and are reported in terms average annual, per FVC, and per dose administered metrics.

### 3.7 Sensitivity analysis

The most critical assumptions made when estimating cost of vaccine delivery relate to coverage, wastage rates, and use of labour at service point. These parameters were varied over an inclusive range while keeping all other inputs at base values. Assumptions on wastage, discount rate, and time to administer the vaccine were generic; country data were used in the baseline for all other inputs varied. Resulting estimates of cost of vaccine delivery are summarized by country in tornado plots.

## 4 Results

### 4.1 Overview of key demographic, coverage, and EPI inputs by country

Differences in country cohorts and the EPI system detailed in Table 2 help interpret the level and variation in costs across countries. The cohort size of Tanzania of 1.7 million infants is about 4 times as large as that of Senegal. There is variation not only in the level of coverage achieved but also in the level of coverage sustained between the doses. In Uganda coverage at third dose is 8 percentage points lower than the first dose; the drop-off is about 3 percentage points in Ghana and Senegal. There is variation in wages at facility level: nearly $800 per month are reported for Kenya, $421 and $115 per month for Tanzania and Burkina Faso, respectively. Interestingly the ordering of countries changes when we look at wages at higher levels of EPI; both at central and district levels highest wages are reported for officers in Ghana. Finally, there are differences in the number of days and number of EPI staff conducting supervision visits across countries; these vary from 0.5 to about 10 days per month with as few as 4 to as many as 9 officers per district involved in supervisory capacity.

### 4.2 Cost of RTS,S immunization

Unless noted, costs are presented for the 6–9 months schedule assuming a vaccine price per dose of $5; estimates for other implementation strategies are reported in Supplementary Tables S2–9. Vaccine introduction costs expressed in terms of financial cost per infant range between $0.76 and $1.72 (Table 3); these costs assume an existing capacity and represent a minimum initial investment needed at country level to introduce the new antigen. Program costs including annualized introduction and annual recurrent costs range from about $21.71 to $22.86 per FVC in financial terms; the range for the economic costs is wider – from $23.11 to $28.28 per FVC across the 6 countries. Annual program costs increase with size of the cohort; cost of procurement accounts for most of these expenditures: vaccines and immunization supplies make up about 95% of financial and 84% of economic costs.

### 4.3 Differences in cost of RTS,S immunization by country

**Fig. 1:**
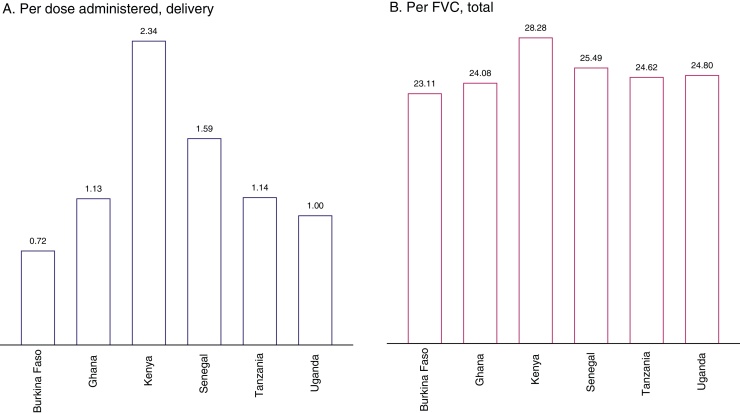
Average annual economic cost of RTS,S immunization deployed via 6–9 months schedule in Burkina Faso, Ghana, Kenya, Senegal, Tanzania, and Uganda (USD, 2013). (A) Bars represent cost of vaccine delivery (program costs net of vaccine and immunization supplies) per dose administered; (B) bars represent total program cost per FVC (child receiving full schedule) including vaccines, immunization supplies, and cost of delivery.

Country differences in cost of vaccine delivery are a function of program design, coverage, EPI structure, number of antigens in the EPI schedule, and prices, the most critical of which is cost of labour. The lowest economic cost of delivery is estimated for Burkina Faso at $0.72 and highest for Kenya – at about $2.34 per dose administered (Fig. 1 A). Over a three-fold difference between the countries is largely driven by wage differentials: compared to Burkina Faso, wages of EPI officers in Kenya are nearly five times higher; the differentials persist across all distribution levels although at lower levels wage differences are smaller (Table 2). In addition, compared to other countries, Kenya has one of the lowest projected coverage rates resulting in a lower denominator and, consequently, a lower base over which the fixed costs, including introduction investment, are allocated. When summarized in terms of economic cost per FVC, variation between countries is much smaller: the lowest estimate is for Burkina Faso at $23.11 and the highest – for Kenya at $28.28 (Fig. 1 B). Convergence in costs at this level is due to differences in coverage between countries; cost per FVC increases steeply with drop-off between doses.

### 4.4 Differences in cost of RTS,S immunization by schedule

**Fig. 2:**
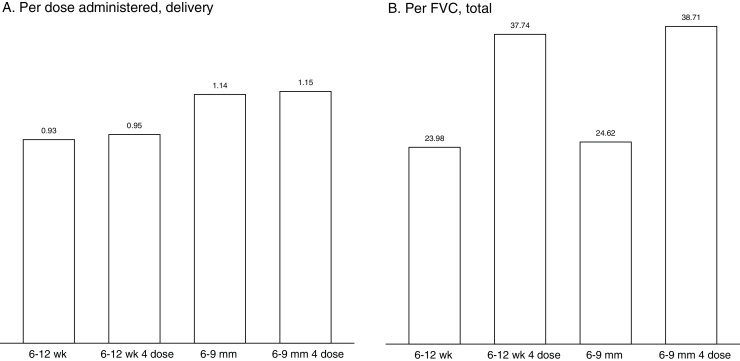
Average annual economic cost of RTS,S immunization in Tanzania by deployment modality (USD, 2013). (A) Bars represent cost of vaccine delivery (program costs net of vaccine and immunization supplies) per dose administered; (B) bars represent total program cost per FVC (child receiving full schedule) including vaccines, immunization supplies, and cost of delivery.

Differences in cost by schedule are similar across the 6 countries; these are illustrated in Fig. 2 for Tanzania. For 6–12 weeks and 6–12 weeks 4 dose implementations, the cost of delivery is lowest; higher costs are estimated for 6–9 months modalities, although at this level differences between strategies are modest. When summarized as cost per FVC, program costs for modalities including doses outside of the routine schedule are significantly higher. The much higher cost per FVC for boosting schedules is again due to coverage assumptions, namely we assumed 80% of 3rd dose coverage for 4th dose, resulting in a lower denominator for these strategies.

### 4.5 Cost drivers of RTS,S immunization

**Fig. 3:**
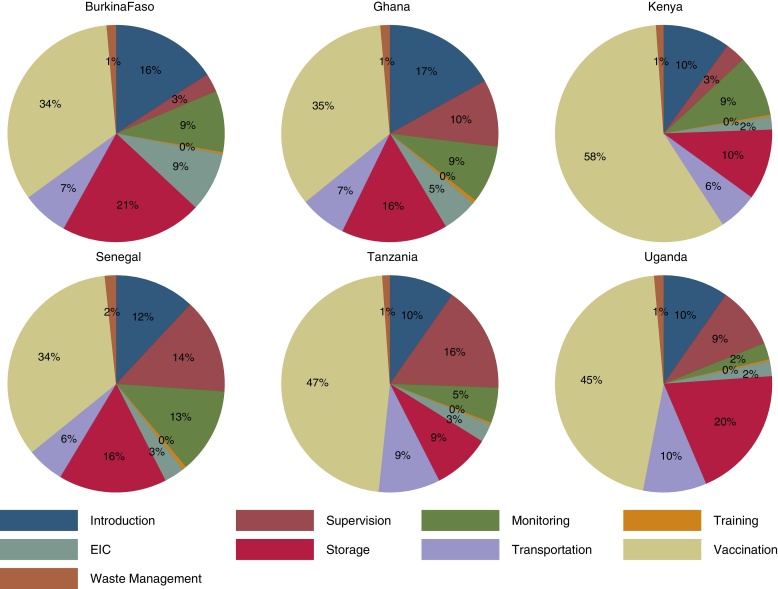
Distribution of average annual economic costs of RTS,S immunization deployed via 6–9 months schedule in Burkina Faso, Ghana, Kenya, Senegal, Tanzania, and Uganda: Service delivery. Pie charts represent the distribution of cost of vaccine delivery (program costs net of vaccine and immunization supplies) by activity; “Vaccination” category covers all costs incurred at point of delivery excluding vaccines and immunization supplies.

Resource requirements for each program component as a proportion of average annual delivery costs are illustrated in Fig. 3. Costs at the facility level associated with the immunization visit account for the largest proportion of total delivery costs. The relative weight of other inputs varies across settings with differences across input categories mainly driven by differences in the structure of EPI program (levels of cold storage, number of staff at each unit, etc.), resource use, and wages. For instance, in Kenya labour heavy vaccination activities account for almost 60% of total delivery costs compared to only about 35% in Burkina Faso where, as discussed, reported wages are significantly lower. Activities such as supervision, monitoring and introduction incorporate heterogeneities in wage structure across countries as well as levels of resource use. The latter is illustrated with supervision activities; in Tanzania supervision accounts for nearly 16% of total program costs based on reported 4–14 days per month devoted to the activity across EPI levels; in contrast, in Kenya and Burkina Faso an average of only 3 days per months are allocated to supervision.

### 4.6 Sensitivity analysis

**Fig. 4:**
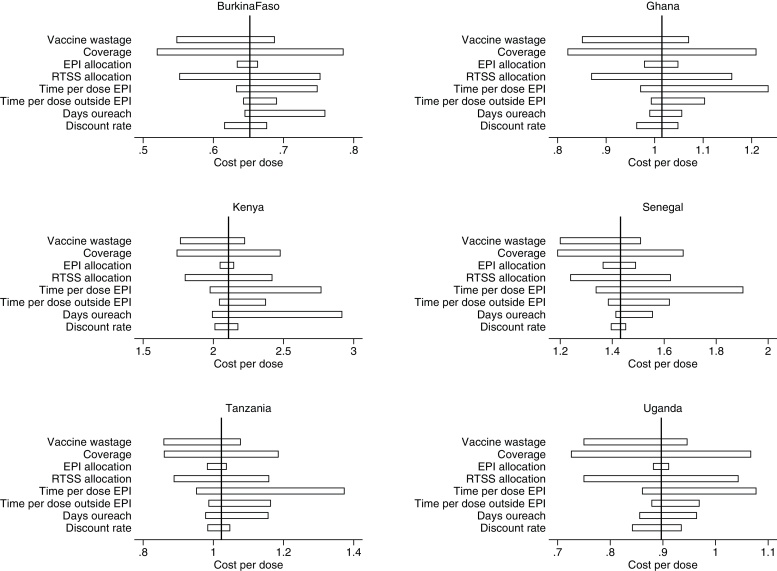
One-way sensitivity analysis on average annual economic cost of RTS,S immunization deployed via 6–9 months schedule in Burkina Faso, Ghana, Kenya, Senegal, Tanzania, and Uganda: Service delivery (USD, 2013). “Vaccine wastage” low value of 5% and a high of 25% are tested. “Coverage” under low scenario is assumed to be 75% of baseline value, at high scenario it is taken to be 125% of baseline. “EPI allocation” and “RTS,S allocation” of shared inputs are scaled down to 75% of baseline value and up to 125% under high scenario. “Time per dose EPI” is varied from 5 min at baseline to 3 and 15 min under low and high scenarios respectively. “Time per dose outside EPI” is varied from 7 min at baseline to 5 and 15 min under low and high scenarios respectively. “Days outreach” under low scenario are set to 2 and to 15 under high scenario. “Discount rate” values of 0% and 10% are evaluated.

Results of the sensitivity analysis show that cost of service delivery changes almost proportionally with immunization coverage (Fig. 4). These are sensitive to assumptions on time to administer the vaccine both within and outside of the EPI schedule; if instead of 5 min to administer an additional vaccine dose [19], [32] 15 min are required, to allow, for instance, for information and incentivisation, service costs would increase by as much as 25%. Use of EPI shared resources including cold chain, vehicles, and program management are among other influential parameters.

## 5 Discussion

Costing an intervention that has not been implemented is a difficult undertaking further complicated by the absence of country policy with respect to its use. This study presented an approach to estimate the prospective costs of RTS,S introduction that can be easily adapted to the specifics of the vaccine presentation, country EPI program, and implementation strategy. One of the main advantages of the methodology is that it can be implemented using routinely collected and publicly available.

Estimates presented should be interpreted in the context of assumptions made on vaccine price, coverage, delivery modality, and data constraints. While we made every attempt to adapt these to country settings, lack of detail on operational aspects of the program might have resulted in a more normative distribution model. Aside from concerns about relevance of activities costed for a given setting, more general concerns about the quality of cMYP reports, borrowing of data and extrapolation across countries to fill in the data gaps introduced additional uncertainty.

One previous study assessed prospective costs of RTS,S introduction via routine EPI in Tanzania [19]; these were estimated using a similar methodology and relying on local data including system capacity use. The incremental cost of vaccine introduction was estimated at $0.66 per dose, which is lower than $0.93 per dose estimated in our study for Tanzania, but comparable given differences in scope. The contribution of the main cost drivers to the vaccine delivery costs were of the same order of magnitude in the two studies. Consistent with these earlier analyses, delivery costs account only for a small proportion of total program costs. At a vaccine price of $5 per dose about 95% of financial and 84% of economic costs are accounted for by vaccines and immunization supplies.

Although comparability with other antigens is limited given differences in vaccine properties, deployment strategies, immunization rate, etc., costs estimated here for RTS,S delivery are similar to other vaccines recently introduced in the region. De la Hoz-Restrepo [33] cited non-vaccine costs associated with the introduction of rotavirus and pneumococcal conjugate vaccines in developing countries at $0.74 (IQR:$0.58–$1.32) and $1.27 (IQR:$0.99–$1.37) per dose, respectively. Griffith [20] estimated the incremental cost of introducing DTwP-hepatitis B-Hib vaccine in Ethiopia at $1.15 per FVC. The estimate is closest in scope to financial vaccine delivery costs presented in this study – $0.90 to $1.91 per FVC across the 6 countries. Klinger et al. [34] reported cost per an additional birth dose of Hepatitis B in Mozambique at $1.46 ($1.27–$2.27). Hutubessy et al. [21] presented costs for HPV introduction in Tanzania using a similar methodology; the deployment strategy for the vaccine required reaching older children outside of the routine EPI delivery schedule with estimated costs of delivery ranging from $1.36 per dose for financial to $3.56 for economic costs. While these findings are yet to be replicated, we note that costs reported here are tied to explicit assumptions on vaccine deployment including labour; as countries decide on the operational strategy for RTS,S introduction estimates could be revised accordingly.

Costs estimated by the study are of interest to a diverse set of stakeholders. At country level these provide baseline values of resources required to introduce and maintain the intervention. Information particularly vital for Gavi graduating countries that will assess not only the additional resource needs for the new antigen but also the increased cost-sharing for provision of immunization for the current schedule and sustainability of the new schedule in the longer term. We show that delivery costs at country level are substantial; for instance, in Tanzania these could range between $0.8 and $4.1 million per year depending on scope of costing and level of spare capacity in the system.

For donors and global institutions supporting immunization programs the analysis presents some initial estimates of resource needs for vaccine introduction; moreover it highlights the extent of resource use beyond procurement supplied by countries when introducing the intervention. Donors, in particular Gavi, might be interested in the cost of particular program components like vaccine introduction investment to gauge the size of introduction grants to support RTS,S in endemic countries. We show that the costs are higher in particular if vaccine is delivered outside of routine schedule and if new visits would be required. For the 6 countries, we estimate introduction costs between $0.76 and $1.72 per infant in the surviving cohort; these are comparable to levels of support currently awarded by Gavi for new vaccine introduction: $0.80 per child for vaccines delivered to infants and $2.40 per girl for HPV vaccine [35].

## Conflict of interest

This work was supported with a research grant from PATH – Malaria Vaccine Initiative – a partner contributing funding, through the Bill and Melinda Gates Foundation, to RTS,S development; KG was supported with this grant. No funding bodies had any role in the study design, data analysis, decision to publish, or preparation of the manuscript. Other contributors have no conflicts of interest to report.

## Figures and Tables

**Table 1:** Activities, assumptions, and inputs for delivery of immunization services.

Stage	Program component	Activity	Activity assumptions	Resource assumptions	Economic costs	Financial costs
Introduction	Planning	- Micro-planning - Development of training materials - Development of EIC materials	- Micro-planning at central level assuming a total of 1 month of preparatory work	- 3 1-day workshops for planning and development of training and IEC materials at central level including national and regional EPI managers (2 from each region)	- Wages - Per-diems - Hotel - Vehicle - Vehicle maintenance and overheads - Consumables - Stationaries - Printed materials - Facility rental	- Per-diems - Hotel - Vehicle - Vehicle maintenance and overheads - Consumables - Stationaries - Printed materials
	Cold store assessment	- Inventory of cold chain, assessment of spare capacity for introduction of new vaccine	- Assessment conducted by EPI cold store staff	- Costed based on estimates from cMYP	Cold store assessment	
	Revision of immunization cards and tally sheets	- Revision of immunization cards and tally sheets to include new vaccine	- Revised cards and tally sheets printed for the target cohort	- 25% reserve stock - Full cost of revised cards and tally sheets printed for the target cohort allocated to RTS,S in the first year, thereafter a proportion allocated to RTS,S	- Printing tally sheets - Printing immunization cards	- Printing tally sheets - Printing immunization cards
	Training	- Training of trainers - Training of regional supervisors - Training of immunization staff	- 5 day training of trainers (2 nurses per district) at central level - 2 day training workshop for regional supervisors at central level - 1 day training of vaccinators at district level (5 nurses from regional and district facilities; 1 from all other levels)	- Start training the year of vaccine introduction - After first year RTS,S specific training integrated into routine EPI training for new staff and refresher courses	- Wages - Per-diems - Hotel - Vehicle - Vehicle maintenance and overheads - Consumables - Stationaries - Printed materials - Facility rental	- Per-diems - Hotel - Vehicle maintenance and overheads - Consumables - Stationaries - Printed materials
	Social Mobilization and IEC	- Launching ceremony at central level - Sensitization meetings at district level - TV advertisement - Radio advertisement - Flyers and posters	- Launching ceremony including 5 speakers, band, volunteers (1/50 attendees), technical staff - IEC meetings at district level involving regional EPI officers(1/district); band, volunteers	- First year of program roll-out, thereafter integrated with routine EPI IEC activities	- Wages - Per-diems - Speaker fees - Band fees - Volunteers - Hotel - Vehicle - Vehicle maintenance and overheads - Consumables - Stationaries - Printed materials - Facility rental - TV, radio advertisement - Flyers, posters	- EPI per-diems - Speaker fees - Band fees - Hotel - Vehicle maintenance and overheads - Consumables - Stationaries Printed materials - TV, radio advertisement - Flyers, posters

Recurrent	Supervision	- Supervision over program implementation	- EPI staff at central and subnational levels	- Number of supervisory visits and staff involved in supervision of EPI including drivers based on cMYP records - Proportion of wages, per-diems, transport costs allocated to RTS,S	- Wages - Per-diems - Vehicle - Vehicle maintenance and overheads	- For deployments including doses outside of routine schedule include EPI wages, per-diems, transportation for the increase in intensity of supervision activities
	Monitoring and program management	- Collecting data on vaccine stock and coverage - Strategic planning Administrative support - Printing of immunization cards and tally sheets - Post introduction evaluation	- NIP staff at central and subnational EPI levels - Post introduction evaluation conducted externally	- Proportion of EPI wages allocated to RTS,S - Proportion of annual printing costs allocated to RTS,S - Post introduction evaluation costed based on estimates from cMYP - Post introduction evaluation annualized over 5 years	- Wages - Tally sheets - Immunization cards - Post introduction evaluation	- Tally sheets Immunization cards - Post introduction evaluation
	Training	- Refresher training and training of new staff	- Integrated into routine EPI training	- Proportion of annual EPI budget for training allocated to RTS,S	- Proportion of annual EPI budget for training allocated to RTS,S	
	Social Mobilization and IEC	- Social Mobilization and IEC on RTS,S	- Integrated into routine EPI Social Mobilization and IEC activities	- Proportion of annual EPI budget for Social Mobilization and IEC	- Proportion of annual EPI budget for Social Mobilization and IEC	
	Procurement	- Procurement of vaccines and supplies	- Procurement of vaccines and supplies through the UNICEF Supply Division	- Including freight and insurance, wastage - UNICEF procurement and handling fee	- Vaccines - Syringes - Alcohol - Cotton wool - Safety boxes - UNICEF handling fee	- Vaccines - Syringes - Alcohol - Cotton wool - Safety boxes - UNICEF handling fee
	Storage	- Storage of vaccines and immunization supplies	- Type of cold store equipment assigned by administrative level based on cold and dry storage volume required	- Storage costs calculated based on volume of vaccines and supplies - Months of storage based on number of deliveries at each level	- Wages - Cold store equipment - Equipment maintenance, overheads - Facility - Facility overheads	- Equipment maintenance, overheads - Facility overheads
	Transportation	- Transport of vaccines and immunization supplies to sub-national stores and health facilities	- Type of vehicle by administrative level - Grossing factors by level and type of equipment used to transport the vaccines	- Fuel, and maintenance based on average distance between administrative units - Transit costs scaled by volume of the vaccine and supplies to be transported by delivery route over vehicle storage capacity of the vehicle	- Wages - Vehicle - Vehicle maintenance and overheads - Cold boxes	- Vehicle maintenance and overheads - Cold boxes annual replacement (20%)
	Vaccination	- Fixed site vaccination - Outreach vaccination	- Vaccines administered by nurses - Outreach from facilities to remote areas by nurses on bikes	- 7 min per dose when administered alone; 5 min when administered with another vaccine - Outreach costed based on number of days conducting outreach activities as per cMYP Immunization office of 20 m^2^	- Wages - Per-diems - Facility - Facility overheads - Furniture - Stationaries - Vaccine carrier - Bicycle	- Facility overheads - Stationaries
	Waste management	- Incineration of syringes and vials	- Incinerator at national, regional, and district facilities - Fire pit at lower level health centers		- Wages - Incinerator, bottle, protective gear crusher - Equipment maintenance and overheads - Bottle crusher - Fuel	- Fuel -Equipment maintenance and overheads

**Table 2:** Overview of key demographic, coverage, and EPI inputs by country.

	Burkina Faso	Ghana	Kenya	Senegal	Tanzania	Uganda
Number of surviving infants^1^	719′218	767′114	1′220′634	453′259	1′716′679	1′450′141
6–9 months coverage dose 1^2^	71%	71%	62%	72%	74%	67%
6–9 months coverage dose 3^2^	66%	68%	57%	69%	68%	59%
Wages nurse	$115	$263	$791	$565	$421	$216
Per-diems outreach nurse	$10	$4	$27	$12	$11	$5
Days outreach nurse	2.5	5	3	3	4	5
Wages EPI manager central level	$563	$2482	$2373	$1131	$1506	$705
Wages EPI manager district level	$428	$1880	$791	$1019	$948	$867
Per-diems EPI managers at district level	$10	$26	$16	$17	$37	$11
Days supervision EPI managers at district level	2	5	2	2–10	4–14	5–10
EPI managers at district level conducting supervision	9	3	7	6	5	4

*Source*: Unless otherwise stated, estimates are extracted from country cMYP costing tool [24–29]; nominal values inflated to 2013 via US GDP deflator. ^1^CIA, The World Factbook (2013). *Surviving infants.* Retrieved from https://www.cia.gov/library/publications/the-world-factbook/;^2^An assumption; coverage in 6–9 months schedule is taken to be 75% of DTP. UNICEF, Child Health, Immunization (2013). *Immunization Coverage by Antigen*. Retrieved from: http://data.unicef.org/child-health/immunization (WUENIC, 2013 revision).

**Table 3:** Summary of average annual costs of RTS,S immunization deployed via 6–9 months schedule in Burkina Faso, Ghana, Kenya, Senegal, Tanzania, and Uganda (USD, 2013).

	Burkina Faso	Ghana	Kenya	Senegal	Tanzania	Uganda
Introduction costs per surviving infant *financial*^a^	$1.04	$1.72	$1.58	$1.59	$0.97	$0.76
Recurrent costs per dose administered *financial*^b^	$6.99	$7.01	$7.17	$7.01	$6.92	$7.08
Total *financial* costs per FVC	$21.82	$21.74	$22.63	$21.71	$21.81	$22.86
Total *economic* costs per FVC	$23.11	$24.08	$28.28	$25.49	$24.62	$24.80
Total procurement costs^c^	$9′903′660	$10′679′279	$14′591′719	$6′447′159	$24′677′812	$18′322′750
Total *financial* costs	$10′361′365	$11′259′059	$15′741′310	$6′789′375	$25′551′472	$19′388′822
Total *economic* costs	$10′970′655	$12′469′923	$19′674′652	$7′972′356	$28′846′864	$21′037′518

a Total financial cost of activities held in the introductory stage (micro-planning, cold chain evaluation, training, etc.) without annualization or discounting. See Table 1 for details.

b Average annual financial costs of running the program, net of introduction costs.

c Cost of vaccines, immunization equipment, and supplies.
