# Supplementary material for: Costing RTS,S introduction in Burkina Faso, Ghana, Kenya, Senegal, Tanzania, and Uganda: A generalizable approach drawing on publicly available data
Source: Vaccine. 2015 Nov 27;33(48):6710–8. doi: 10.1016/j.vaccine.2015.10.079 (PMC5357730; doi:10.1016/j.vaccine.2015.10.079)
Supplement: Supplementary file 1 [file mmc1.docx]

**Supplementary File S1: Assumptions for capacity scale-up**

**Expanding cold chain storage**

Based on the cold store assessment detailed above, allowances are made for additional cold chain equipment to meet the capacity required for the new EPI schedule including the RTS,S vaccine. We factor in, by distribution level, the cost of corresponding cold chain equipment including installation and supplementary items (i.e. thermometer, generator, freezer); the cost of cold chain equipment and related overheads are calculated on per cubic meter (/m3) per month basis and are multiplied by the required capacity and length of storage to estimate the total costs of resource expansion. These expenditures related to additional capacity are included in both average and marginal analyses.

**Expanding labour**

For purposes of this evaluation we consider labour to be fixed and while shortage of qualified medical staff is noted across the EPI levels in the three countries evaluated expansion of labour is not accommodated in this analysis. The assumption, however, could be easily relaxed. For settings where it is feasible to expand medical staff to accommodate the RTS,S, cost of a paper advertisement (1 month), review of candidates and subsequent interviews (1 work day), and a 5 day training could be costed proportional to the number of newly hired staff. Costs of these additional vaccinators or EPI officers are to be included in both average and marginal analyses.

**Expanding transportation**

Capacity to accommodate transportation of vaccines and supplies is evaluated by distribution level. Based on identified shortages, vehicles similar to those already in use by the EPI, at each administrative level are costed. Similarly to cold chain expansion, cost of vehicles is calculated to per cubic meter per kilometre (/m3/km) basis and scaled to accommodate identified capacity needs. That is, cold chain and transport costs are assumed to be continuous variables, and are not costed in natural units of vehicles, cold boxes, etc. Costs of these additional vehicles are included in both average and marginal analyses.

**Expanding waste management**

Additional incinerators at central, regional, and district levels to accommodate waste management of RTS,S and additional fuel for fire pits are costed in settings where expansions in waste management capacity are required.

**Supplementary File S3: Algorithm for calculating cost of immunization**

*The algorithm and formulas defined here produce estimates of economic costs; these could be modified to generate estimates of financial costs by editing resource lists as defined in Table1 and removing the discounting in valuation of capital inputs*.

Costs of vaccine introduction are described by the following formula:

$\boldsymbol{TC=F+M+E+O+P+S+T+I+G}$, where **(1)**

*F* is cost of introduction activities

*M* is cost of activities related to social mobilization and EIC

*E* is cost of training

*O* is cost of supervision

*V* is cost of procurement

*P* is cost of program management, monitoring and surveillance activities

*S* is cost of storage

*T* is cost of transportation

*I* is cost of immunization

*G* is cost of waste management

**Introduction activities (*F*)**

Total introduction costs (*F*) could be represented as follows:

$\boldsymbol{F=P+A+F+Tt+Ts+Tv+M},$ where **(2)**

*P* is cost of activities related to planning vaccine introduction including mirco-planning, development of training and EIC materials

*A* represents expenditures related to cold-chain assessment

*F* revision of immunization cards and tally sheets

*T_t_* captures expenditures related to training of trainers

*T_s_* captures expenditures related to training of supervisors

*T_v_* captures expenditures related to training of vaccinators

*M* captures expenditures related to social mobilization and IEC activities

Activities related to planning vaccine introduction (*P*) can be described by the following formula:

$\boldsymbol{P=W+T+H+F+C+S}$, where **(2.1)**

*W* is wages including per-diems of EPI staff at all levels involved in planning activities

*T* is cost of transportation

*H* is hotel costs

*F* is cost of facility rental

*C* is cost of consumables

*S* is cost of stationaries

Wages of EPI workers (*W*) are described by the following formula:

$\boldsymbol{W=}\sum_{\boldsymbol{i=1}}^{\boldsymbol{n}} \frac{\boldsymbol{W}_{\boldsymbol{1}\boldsymbol{i}}}{\boldsymbol{20}}\boldsymbol{*}\boldsymbol{d}_{\boldsymbol{1}\boldsymbol{i}}\boldsymbol{+}\left[ \sum_{\boldsymbol{i=1}}^{\boldsymbol{m}} \left( \frac{\boldsymbol{W}_{\boldsymbol{2}\boldsymbol{i}}}{\boldsymbol{20}}\boldsymbol{+}\boldsymbol{q}_{\boldsymbol{2}\boldsymbol{i}} \right) \right]\boldsymbol{*}\boldsymbol{s}_{\boldsymbol{2}}\boldsymbol{*}\boldsymbol{d}_{\boldsymbol{2}}$, where **(2.1.1)**

*W_1i_* is monthly wages of EPI officer *i* at central level

*d_1i_* is number of days allocated for planning activities involving regional managers (i.e. workshops for planning and development of training and EIC materials)

*W_2i_* is monthly wages of EPI officer *i* at regional level

*q_2i_* is per-diem for EPI staff *i* at regional level

*s_2_* is number of regional stores

*d_2_* is number of days allocated for planning activities involving regional managers (i.e. workshops for planning and development of training and EIC materials)

*In the paper we assumed that all central level staff in managerial positions (excluding custodial staff, drivers, etc.) as captured by the cMYP will spend 30 days planning the vaccine introduction (d_1i_=30, for all i=1 to n). Further 3 1-day workshops with national and regional staff are assumed to be carried out for planning purposes (d_2_ =3). We assume 2 regional level EPI representatives to take part in these planning meetings (i.e. one Regional Health Officer and one Medical Officer)(m=2).*

Cost of transportation (*T*) for planning meetings for regional EPI staff is calculated as:

$\boldsymbol{T=W+V+F+M}$, where **(2.1.2)**

*W* is driver wages and per-diems

*V* is cost of vehicle

*F* is cost of fuel

*M* is cost of maintenance

Driver wages and per-diems (*W*) are calculated as:

$\boldsymbol{W=}\left( \frac{\boldsymbol{w}_{\boldsymbol{2}}}{\boldsymbol{20}}\boldsymbol{+}\boldsymbol{q}_{\boldsymbol{2}} \right)\boldsymbol{*}\boldsymbol{dd}_{\boldsymbol{2}}\boldsymbol{*}\boldsymbol{s}_{\boldsymbol{2}}$, where **(2.1.2.1)**

*w_2_* is wages of drivers at regional level

*q_2_* is driver per-diems at regional level

s_2_ is number of stores at regional level

*dd_2_* is number of workshops including regional managers

Vehicle operational cost (*V*) is in turn calculated as:

$\boldsymbol{V=}\frac{\boldsymbol{M}}{\boldsymbol{U}}\boldsymbol{*}\boldsymbol{D}_{\boldsymbol{2}}\boldsymbol{*}\boldsymbol{s}_{\boldsymbol{2}}\boldsymbol{*}\boldsymbol{dd}_{\boldsymbol{2}},$where **(2.1.2.2)**

*M* is cost of vehicle

*U* is vehicle ULY in km

*D_2_* is RT distance from regional store to central facility

*s_2_* is number of regional stores

*dd_2_* number of planning meetings

Fuel costs (*F*) are estimated as follows:

$\boldsymbol{F=f*C*}\boldsymbol{D}_{\boldsymbol{2}}\boldsymbol{*}\boldsymbol{s}_{\boldsymbol{2}}\boldsymbol{*}\boldsymbol{dd}_{\boldsymbol{2}}$, where  **(2.1.2.3)**

*f* is cost of fuel per km

*C* is vehicle fuel consumption per km

*D_2_* is RT distance from regional store to central facility in km

*s_2_* is number of regional stores

*dd_2_* number of planning meetings

Maintenance costs (*M*) are assumed to be 15% of total fuel costs (*F*):

$\boldsymbol{M=F*.15}$  **(2.1.2.4)**

Hotel costs (*H*) are estimated as:

$\boldsymbol{H=h*}\boldsymbol{n}_{\boldsymbol{2}}\boldsymbol{*}\boldsymbol{s}_{\boldsymbol{2}}\boldsymbol{*}\boldsymbol{d}_{\boldsymbol{2}}\boldsymbol{*}\boldsymbol{dd}_{\boldsymbol{2}}$**,** where **(2.1.3)**

*h* is hotel cost per night

*n_2_* is number of EPI officers per regional level attending the meetings

*s_2_* is number of regional stores

*d_2_* is number of days per workshop

*dd_2_* number of planning meetings

Facility rental costs (*F*) are calculated assuming 2m2/ per attendee:

$\boldsymbol{F=f*}\left( \boldsymbol{n}_{\boldsymbol{1}}\boldsymbol{+}\boldsymbol{n}_{\boldsymbol{2}}\boldsymbol{*}\boldsymbol{s}_{\boldsymbol{2}} \right)\boldsymbol{*2*}\boldsymbol{d}_{\boldsymbol{2}}\boldsymbol{*}\boldsymbol{dd}_{\boldsymbol{2}}$**,** where  **(2.1.4)**

*f* is facility rental per m2

*n* is number of EPI officers from central level participating in planning meetings

*m* is number of EPI staff per regional level participating in planning meetings

*s_2_* is number of regional stores

*d_2_* is number of days per workshop

*dd_2_* number of planning meetings

Cost of consumables (*C*) for planning meetings is estimated as:

$\boldsymbol{C=q*}\left( \boldsymbol{n+m*}\boldsymbol{s}_{\boldsymbol{2}} \right)\boldsymbol{*}\boldsymbol{d}_{\boldsymbol{2}}\boldsymbol{*}\boldsymbol{dd}_{\boldsymbol{2}}$**,** where **(2.1.5)**

q is cost of consumables per person (i.e. snacks, refreshments, etc.)

*n* is number of EPI officers from central level participating in planning meetings

*m* is number of EPI officers from regional level participating in planning meetings

*s_2_* is number of regional stores

*d_2_* is number of days per workshop

*dd_2_* number of planning meetings

*In the paper we make an allowance for consumables equal to 1/3 of per-diem at central level (*$q=\frac{q_{1}}{3}$*).*

Cost of stationaries (*S*) is calculated as:

$\boldsymbol{S=(z+p)*}\left( \boldsymbol{n+m*}\boldsymbol{s}_{\boldsymbol{2}} \right)\boldsymbol{*}\boldsymbol{dd}_{\boldsymbol{2}}$**,** where **(2.1.6)**

*z* is cost per pens per person

*p* is cost of paper allowance per person

*n* is number of EPI officers from central level participating in planning meetings

*m* is number of EPI officers from regional level participating in planning meetings, per region

*s_2_* is number of regional stores

*dd_2_* number of planning meetings

Cost of cold chain assessment (*A*) is taken directly from cMYP document; no formulas were defined for this component.

Cost of printing tally sheets (monthly/ 12 per year) and immunization cards (*P*) including a 25% buffer is calculated as:

$\boldsymbol{F=}\boldsymbol{(f}_{\boldsymbol{c}}\boldsymbol{*B+}\boldsymbol{f}_{\boldsymbol{s}}\boldsymbol{*}\boldsymbol{s}_{\boldsymbol{4}}\boldsymbol{*12)*1.25}$, where **(6.2)**

*f_c_* is cost of printing an immunization card

*B* is cohort size

*f_s_* is cost of printing a tally sheet

*s_4_* is number of health facilities providing immunization

Training of trainers (*T_t_*) is captured by the following formula:

$\boldsymbol{T}_{\boldsymbol{t}}\boldsymbol{=W+T+H+F+C+S+P}$, where **(2.2)**

*W* is wages including per-diems of staff trained

*T* is cost of transportation for trainers to central facility

*H* is hotel costs for trainer accommodations

*F* is facility rental costs

*C* is cost of consumables

*S* is cost of stationaries

*P* is cost of printed materials

Formula for wages of trainers (*W*) is based on the assumption that medical staff from district levels are trained in this capacity at regional level by the regional staff and is calculated as follows:

$\boldsymbol{W=}\left[ \sum_{\boldsymbol{i=1}}^{\boldsymbol{n}} \left( \frac{\boldsymbol{w}_{\boldsymbol{3}\boldsymbol{i}}}{\boldsymbol{20}}\boldsymbol{+}\boldsymbol{q}_{\boldsymbol{3}\boldsymbol{i}} \right)\boldsymbol{*}\boldsymbol{s}_{\boldsymbol{3}}\boldsymbol{+}\sum_{\boldsymbol{i=1}}^{\boldsymbol{m}} \frac{\boldsymbol{W}\boldsymbol{2}\boldsymbol{i}}{\boldsymbol{20}}\boldsymbol{*}\boldsymbol{s}_{\boldsymbol{2}} \right]\boldsymbol{*}\boldsymbol{d}_{\boldsymbol{3}}$**,** where  **(2.2.1)**

*k* is number of trainers per district

*w_3_* is monthly wages of medical staff *i* at district level

*q_3_* is per-diem of medical staff *i* at district level

*s_3_* is number of district stores in a country

*w_2_* is monthly wages of medical staff *i* at regional level

*q_2_* is per-diem of medical staff *i* at regional level

*s_2_* is number of regional stores in a country

*d_3_* is number of days per workshop

*In the paper we assume that 2 Public Health Nurses (or equivalent as defined by cMYP EPI district staff structure) per district are trained as trainers at regional level(k=2); training is conducted by the central EPI staff whose wages are covered under general planning activities (Equation 1.1.1). Training is carried out over 5 days (d_3_=5).*

T, H, F, C, and S are calculated following the formulas represented by Equations 2.1.2-5 respectively. Quantities are updated with assumptions on number of for trainers at district for regional level and respective distances. Facility and consumables costs include both the central and district staff; central staff required to train district workers is assessed assuming 50 trainees per trainer.

Cost of printed materials (*P*) is estimated as follows:

$\boldsymbol{P=f*n*(k*}\boldsymbol{s}_{\boldsymbol{3}}\boldsymbol{+}\frac{\boldsymbol{k*}\boldsymbol{s}_{\boldsymbol{3}}}{\boldsymbol{50}}\boldsymbol{)}$**,** where **(2.2.2)**

*f* is cost of printing per sheet (A4)

*n* is number of sheets in the training manual

*k* is number of trainees per district

*s_3_* is number of districts

Training of regional supervisors (*T_s_*) is assumed to be carried out at central level and is captured by the following formula:

$\boldsymbol{Ts=W+T+H+F+C+S+P}$, where **(2.3)**

*W* is wages including per-diems of staff trained

*T* is cost of transportation for regional supervisors to central facility

*H* is hotel costs for supervisor accommodations

*F* is facility rental costs

*C* is cost of consumables

*S* is cost of stationaries

*P* is cost of printed materials

W, T, H, F, C, S, and P are calculated based on the formulas as defined above; quantities and distances are adjusted to reflect the staff requirements and region to central stores distances.

Cost of training vaccinators (*Tv*) is captured by the following formula:

$\boldsymbol{Tv=W+T+F+C+S+P}$, where **(4)**

*W* is cost of EPI staff wages

*T* is cost of transportation to district facilities

*F* is cost of facility rental

*C* is cost of consumables

*S* is cost of stationaries

*P* is cost of printed materials

*W, T, F, C, S,* and *P* are described by formulas detailed above. Assumptions on quantities are updated as per respective scenario.

*In this paper we assume that training of health care workers involved in immunizations is held at district level. 5 workers are trained per central, regional and district facilities; 1 vaccinator for all other levels. Training is conducted by district EPI staff and lasts 1 day. We assume that informational booklets of 100 pages are printed and distributed to facilitate the training. Training is conducted the year of vaccine introduction, thereafter RTS,S is integrated into the routine EPI training and refresher courses- a fraction of these annual costs based on cMYP assessment is attributed to the RTS,S vaccine.*

Total cost of EIC activities and social mobilization can be expressed as:

$\boldsymbol{M=L+S+R}$, where **(3)**

*L* is cost of launching ceremony at the central level

*S* is cost of community sensitization meetings at district levels

*R* is costs of advertising including TV and print media

Formula describing activities and costs related to launching ceremonies (L) are defined based on the scenario detailed in Table1. Key inputs include speaker fees, band fees, volunteer per-diems, payments for support technical staff, facility rental, consumables for support staff, an allowance for miscellaneous expenses, and cost of printed media.

$\boldsymbol{L=W+F+C+A+P}$, where **(3.1)**

*W* is wages, speaker fees, volunteer per-diems

*F* is facility rental costs

*C* is cost of consumables

*A* is an allowance for miscellaneous expenses

*P* is cost of printed media

EPI wages, speaker fees, volunteer per-diems (*W*) and related labour costs are estimated as:

$\boldsymbol{W=}\sum_{\boldsymbol{i=1}}^{\boldsymbol{m}} \boldsymbol{w}_{\boldsymbol{i}}\boldsymbol{*}\boldsymbol{n}_{\boldsymbol{i}}\boldsymbol{*}\boldsymbol{d}_{\boldsymbol{i}}$, where **(3.1.1)**

*i* is unit of labour involved in launching ceremony

*w_i_* is daily payment for services rendered

*n_s_* is number of staff

*d_i_* is number of days involved in preparation for or running the launching ceremony

*F, C*, and *P* are estimated as described by formulas captured by Equations above updating quantity assumptions based on the relevant scenario.

*In this paper we assume 5 external speakers to be presenting at the launching ceremony; 1 volunteer per 50 attendees; a band; and 10 technical workers to support the event. Involvement of central EPI workers is assumed to be costed under general planning allowance. Band fee taken to be equivalent to monthly wages of a Public Health Nurse. Assumptions regarding the number of attendees at the launching ceremony are made for each country based on cohort size in the capital. An allowance of 1,000 USD is assumed for miscellaneous expenditures. Cost of printed media is estimated assuming 2 printed sheets per attendee.*

Expenditures related to community mobilization at district level (S) are defined following the scenario detailed in Table 1 in main text. Cost inputs include wages and per-diems for regional staff, speaker fees, hotel and transportation for regional staff, band fees, volunteer per-diems, facility rental, consumables, an allowance for miscellaneous expenses, and cost of printed media.

$\boldsymbol{S=}\left( \boldsymbol{W+H+T+}\boldsymbol{f}_{\boldsymbol{b}}\boldsymbol{+}\boldsymbol{n}_{\boldsymbol{v}}\boldsymbol{*}\boldsymbol{f}_{\boldsymbol{v}}\boldsymbol{+F+C+A+P} \right)\boldsymbol{*}\left( \boldsymbol{s}_{\boldsymbol{3}}\boldsymbol{-1} \right)\boldsymbol{*}\boldsymbol{d}_{\boldsymbol{3}}$, where **(3.2)**

*W* is wages and per-diems for regional staff conducting EIC activities at regional level

*H* is hotel costs for supervisor accommodations

*T* is cost of transportation for regional supervisors to central facility

*f_b_* is band fees

*n_v_* is number of volunteers

*f_v_* is volunteer per diems

*F* is facility rental costs

*C* is cost of consumables

*A* is an allowance for miscellaneous expenses

*P* is cost of printed materials

*s_3_* is number of district stores

*d_3_* is number of days over which EIC meetings take place in each district

*In this paper we assume 3 regional EPI officers travelling to districts to conduct EIC related activities. Band fees are proxied with 50% of PHI monthly wages. Number of volunteers is based assuming 1 volunteer per 50 attendees, volunteer per-diems as based on PHI per-diems for outreach activities. Assumptions regarding the number of attendees at the district sensitization meetings are made for each country based on district cohort size (See Supplemental Information File S3). An allowance of 250 USD is assumed for miscellaneous expenditures. Cost of printed media is estimated assuming 2 printed sheets per attendee. We assume one 1-day meeting per district (d_3_=1).*

W, F, C, and P are estimated as described by formulas detailed above updating quantity assumptions based on the relevant scenarios.

Cost of advertisement (R) is calculated as follows:

$\boldsymbol{R=}\boldsymbol{R}_{\boldsymbol{tv}}\boldsymbol{+}\boldsymbol{R}_{\boldsymbol{r}}\boldsymbol{+}\boldsymbol{R}_{\boldsymbol{p}}\boldsymbol{+P}$, where **(3.3)**

*R_tv_* is cost of TV advertisement

*R_r_* is cost of radio segments

*R_p_* is cost of advertisement in newspapers

*P* is cost of print media (i.e. posters, flyers, leaflets)

Cost of TV advertisement (*R_tv_*) is described by a formula:

$\boldsymbol{R}_{\boldsymbol{tv}}\boldsymbol{=}\boldsymbol{f}_{\boldsymbol{tv}}\boldsymbol{*}\boldsymbol{t}_{\boldsymbol{tv}}\boldsymbol{*}\boldsymbol{n}_{\boldsymbol{tv}}$**,** where  **(3.3.1)**

*f_tv_* is rate per minute of TV advertisement (loaded rate including cost of development, production)

*t_tv_* is length of the segment

*n_tv_* is number of segments per year

*R_r_*, *R_p_* is captured by the same formula described above.

*In this paper we assume length of TV and radio advertisements of 30 seconds (t_tv_, t_r_=.5). An intense campaign of 3 daily messages on national programs for the first 3 months(20 days/ month), the 2 daily messages for another 3 months, and once daily reminders through the end of the year. Thereafter IEC messages are delivered in the context of routine EPI communications. For advertisement in newspapers estimates are based on ¼ page segment to be run in 3 major outlets for one year.*

Cost of print materials (*P*) is described by a formula:

$\boldsymbol{P=}\boldsymbol{f}_{\boldsymbol{p}}\boldsymbol{*}\boldsymbol{s}_{\boldsymbol{4}}\boldsymbol{*1.25+}\boldsymbol{f}_{\boldsymbol{f}}\boldsymbol{*B*1.25}$, where **(3.3.2)**

*f_p_* is cost of print poster 1m by 1m

*s_4_* is number of health facilities

*f_f_* is cost of printing a flyer(A4)

*B* is country birth cohort size

**Supervision *(O)***

Cost of supervision (*O*) over vaccine introduction is described as:

$\boldsymbol{O=W+T}$, where **(5)**

*W* is wages of central, regional, and district staff involved in EPI supervisory capacity including per-diems

*T* is cost of transportation

Wages for supervisory activities related to the new vaccine are costed by first calculating the total cost of supervisory activities conducted by the EPI staff and then allocating a fraction of these costs to RTS,S. The assumption is that amount of time allocated to a given antigen for supervisory activities is proportional to the number of doses. The calculation is represented by the following formula:

$\boldsymbol{W=}\left[ \sum_{\boldsymbol{l=1}}^{\boldsymbol{e}} \sum_{\boldsymbol{i=1}}^{\boldsymbol{m}} \boldsymbol{(}\frac{\boldsymbol{W}_{\boldsymbol{li}}}{\boldsymbol{20}}\boldsymbol{+}\boldsymbol{q}_{\boldsymbol{li}}\boldsymbol{)*}\boldsymbol{d}_{\boldsymbol{li}} \right]\boldsymbol{*}\frac{\boldsymbol{n}}{\boldsymbol{N}}$, where **(5.1)**

*l* is level of EPI system (i.e. central, regional, district)

*i* is EPI staff at level *l* involved in supervisory activities

*W_li_* is monthly wages of EPI staff *i* at level *l* involved in supervisory activities

*q_li_* is per-diem for EPI staff *i* at level *l* involved in supervisory activities

*d_li_* is number of days EPI staff *i* at level *l* is conducting supervisory visits per year

*n* is number of doses in RTS,S schedule

*N* is number of vaccine doses in EPI schedule

*T* is estimated as described by the formula detailed above; travel distances are assumed to be 100km per day RT; as with labour costs wages are allocated to RTS,S based on the ratio of number of doses required for RTS,S over total number of doses in new EPI schedule.

**Monitoring, and Program Management**

Cost of activities related to monitoring and general program management are captured by the following formula:

$\boldsymbol{M=W+P+A}$, where **(6)**

*W* is wages of EPI staff at central, regional, district levels

*P* is cost of printing tally sheets and immunization cards

*A* is cost of post introduction evaluation

Wages for monitoring and program management (*W*) activities are calculated net of wages attributed to supervisory activities and allocated to RTS,S based on the ratio of number of doses required for RTS,S over total number of doses in new EPI schedule. The following formula details this calculation:

$\boldsymbol{W=}\left[ \sum_{\boldsymbol{l=1}}^{\boldsymbol{e}} \sum_{\boldsymbol{i=1}}^{\boldsymbol{m}} \boldsymbol{(}\boldsymbol{W}_{\boldsymbol{li}}\boldsymbol{-(}\frac{\boldsymbol{W}_{\boldsymbol{li}}}{\boldsymbol{20}}\boldsymbol{*}\frac{\boldsymbol{d}_{\boldsymbol{li}}}{\boldsymbol{12}}\boldsymbol{)} \right]\boldsymbol{*12*}\frac{\boldsymbol{n}}{\boldsymbol{N}}$**,** where **(6.1)**

*l* is level of EPI system (i.e. central, regional, district)

*i* is EPI staff at level *l* involved in supervisory activities

*W_li_* is monthly wages of EPI staff *i* at level *l* involved in supervisory activities

*d_li_* is number of days EPI staff *i* at level *l* is conducting supervisory visits per year

*n* is number of doses in RTS,S schedule

*N* is number of vaccine doses in EPI schedule

Cost of post-introduction evaluation (*A*) is obtained directly from cMYP; no formulas are defined for this cost category.

**Social mobilization and IEC (*M*)**

*We assume that after vaccine is rolled out activities related to social mobilization and IEC for RTS,S are integrated within the routine service provision. A proportion of annual budget is allocated to RTS,S.*

**Training (*E*)**

*We assume that after vaccine is rolled out RTS,S related training is integrated into training of new EPI staff and refresher courses. A proportion of annual budget is allocated to RTS,S.*

**Procurement (*V*)**

Procurement of immunization supplies is described by the following formula:

$\boldsymbol{V=}\left[ \sum_{\boldsymbol{c=1}}^{\boldsymbol{n}} \boldsymbol{f}_{\boldsymbol{c}}\boldsymbol{*}\boldsymbol{n}_{\boldsymbol{c}}\boldsymbol{*}\frac{\boldsymbol{1}}{\left( \boldsymbol{1-}\boldsymbol{W}_{\boldsymbol{c}} \right)} \right]\boldsymbol{*}\left( \boldsymbol{1+F} \right)\boldsymbol{*B*C}$, where **(7)**

*c* is immunization supplies (i.e. vaccine, syringes, cotton, etc.)

*f_c_* is cost of immunization supply c

*n_c_* is number of units of immunization supplies c required per dose

*w_c_* is wastage for immunization supply c

*F* is freight

*B* is birth cohort

*C* is proportion of birth cohort immunized; if multiple doses are required C is the sum of expected coverage for each dose

*In this paper, given our focus on SSA countries we assume that immunization supplies are purchased through the UNICEF Supply Division; thus an additional UNICEF procurement fee is added.*

**Storage (*S*)**

Cost of cold and dry storage (*S*) for vaccines and immunization supplies is captured by the following formula:

$\boldsymbol{S=W+E+F}$, where **(8)**

*W* is wages of cold chain personnel across all storage levels

*E* is cost of cold chain equipment including maintenance and overheads

*F* is cost of facility including overheads

Wages of cold chain staff are calculated as:

$\boldsymbol{W=}\left[ \sum_{\boldsymbol{l=1}}^{\boldsymbol{n}} \sum_{\boldsymbol{i=1}}^{\boldsymbol{m}} \boldsymbol{w}_{\boldsymbol{li}}\boldsymbol{*}\boldsymbol{n}_{\boldsymbol{li}}\boldsymbol{*}\boldsymbol{a}_{\boldsymbol{l}}\boldsymbol{*}\boldsymbol{s}_{\boldsymbol{l}}\boldsymbol{*}\boldsymbol{p}_{\boldsymbol{l}} \right]\boldsymbol{*}\frac{\boldsymbol{v}}{\boldsymbol{V}}$, where **(8.1)**

*l* is level of storage facility (i.e. central, regional, district, etc.)

*i* is staff unit at storage facility at level *l*

*w_li_* is monthly wages of staff *i* at storage facility level *l*

*n_li_* is number of staff *i* at storage facility level *l*

*a_l_* is number of months vaccines and immunization are stored at level *l*

*s_l_* is number of stores at level *l*

*p_l_* is facility usage for EPI related activities at level *l*

*v* is volume of RTS,S vaccine

*V* is total volume of all vaccines in EPI schedule

Cost of storage and related equipment (*E*) including maintenance (5%) and overheads is captured by the following formula:

$\boldsymbol{E=}\sum_{\boldsymbol{l=1}}^{\boldsymbol{m}} \left[ \left( \boldsymbol{E}_{\boldsymbol{l}}^{\boldsymbol{c}}\boldsymbol{*}\boldsymbol{v}_{\boldsymbol{l}}\boldsymbol{*1.05+}\sum_{\boldsymbol{e=1}}^{\boldsymbol{z}} \boldsymbol{E}_{\boldsymbol{le}}\boldsymbol{*}\boldsymbol{n}_{\boldsymbol{le}}\boldsymbol{*}\boldsymbol{p}_{\boldsymbol{l}}\boldsymbol{*}\frac{\boldsymbol{v}}{\boldsymbol{V}}\boldsymbol{*1.05+}\sum_{\boldsymbol{i=1}}^{\boldsymbol{q}} \boldsymbol{C}_{\boldsymbol{li}}\boldsymbol{*}\boldsymbol{n}_{\boldsymbol{le}}\boldsymbol{*f*t*}\boldsymbol{p}_{\boldsymbol{l}}\boldsymbol{*}\frac{\boldsymbol{v}}{\boldsymbol{V}} \right)\boldsymbol{*}\boldsymbol{s}_{\boldsymbol{l}}\boldsymbol{*}\boldsymbol{a}_{\boldsymbol{l}} \right]$ **(8.2)**

where

*l* is level of storage facility

*E^c^_l_* is annualized and discounted cost of cold chain equipment *c* at level *l* in m3

*v_l_* is volume of vaccines stored at level *l* in m3

*g_l_* is grossing factor for cold chain equipment used at store level *l*

*e* is type of supplementary equipment other than cold chain

*E_le_* is annualized and discounted cost of equipment unit *e* used at store level *l*

*n_le_* is number of equipment units *e* used at store level *l*

*p_l_* is percent time equipment unit *e* is used for EPI at level *l*

*v* is volume of RTS,S vaccine

*V* is total volume of all vaccines in EPI schedule

*i* is type of equipment including cold chain used at store level *l*

*C_li_* is energy consumption of equipment unit *i* used at level *l* (i.e. electricity, diesel)

*f* is cost per unit of energy source

*t* is length of operation per month

*s_l_* is number of stores at level l

*a_l_* is length of storage at store level *l*

Where *v_l_* is calculated as:

$\boldsymbol{v}_{\boldsymbol{l}}\boldsymbol{=}\frac{\boldsymbol{v}}{\boldsymbol{n}}\boldsymbol{*}\frac{\boldsymbol{1}}{\boldsymbol{1-w}}\boldsymbol{*b*}\boldsymbol{g}_{\boldsymbol{l}}\boldsymbol{*B*C}$, where **(8.2.1)**

*v* is volume per vaccine vial

*n* is number of doses per vial

*w* is vaccine wastage rate

*b* is bulking factor

*g_l_* is grossing factor for cold chain storage at level l

*B* is cohort size

*C* is proportion of birth cohort immunized; if multiple doses are required C is the sum of expected coverage for each dose

*In this paper, we made generic assumptions on types of cold chain and related equipment used at each store level; these are based on cohort size and expected volume of vaccines to be stored at each level. See Supplemental Information File S3 for country assumptions. Grossing factors are calculated for each type of cold chain equipment based on the dimensions specified for equipment selected. Additionally, cost of a power generator was added to each of the central, regional and district stores (1 unit per store); and one freezer at each of the district level stores. It was further assumed that cold chain equipment is powered with electricity; diesel is costed as source of power for the power generator.*

Facility costs (*F*) are calculated for central, regional, and district levels and are represented by the following formula:

$\boldsymbol{F=}\sum_{\boldsymbol{l=1}}^{\boldsymbol{m}} \left( \left( \boldsymbol{v}_{\boldsymbol{l}}\boldsymbol{+}\boldsymbol{vs}_{\boldsymbol{l}} \right)\boldsymbol{/}\boldsymbol{h}_{\boldsymbol{l}} \right)\boldsymbol{*}\boldsymbol{f}_{\boldsymbol{l}}\boldsymbol{*}\boldsymbol{a}_{\boldsymbol{l}}$, where **(8.3)**

*l* is level of storage facility (i.e. central, regional, district, etc.)

*v_l_* is volume of vaccines stored at level *l* in m3

*vs_l_* is volume of immunization supplies stored at level *l* in m3

*h_1_* is height of the storage unit at store level *l*

*f_1_* is annualized and discounted cost of storage facility rental at level l per month per m2

*a_1_* is number of months supplies are stored at store level l

Where volume for dry storage of immunization supplies are computed following the basic formula as outlined above; no grossing factors are assumed for dry storage.

**Transportation (*T*)**

Cost of transporting vaccines and immunization supplies from central levels to point of delivery can be represented as:

$\boldsymbol{T=W+V+F+M+S}$, where **(9)**

*W* is wages including per-diems of drivers

*V* is cost of vehicles including maintenance

*F* is cost of fuel

*M* is cost of vehicle maintenance

*S* is cost of cold boxes

Wages (*W*) of drivers and per-diems associated with transport of vaccines and immunization supplies are estimated via the following formula:

$\boldsymbol{W=}\sum_{\boldsymbol{l=1}}^{\boldsymbol{m}} \boldsymbol{d}_{\boldsymbol{l}}\boldsymbol{*}\left( \frac{\boldsymbol{W}_{\boldsymbol{l}}}{\boldsymbol{20}}\boldsymbol{+}\boldsymbol{q}_{\boldsymbol{l}} \right)\boldsymbol{*}\boldsymbol{Z}_{\boldsymbol{l}}$, where **(9.1)**

*l* is level of storage facility (i.e. central, regional, district, etc.)

*d_1_* is days spent delivering vaccines and immunization supplies by driver at level *l* per year

*W_l_* is driver monthly wages at level *l*

*q_l_* is driver per-diems at level *l*

*Z* is scaling factor

Where days spent delivering vaccines and immunization supplies (*d_l_*) by driver at level *l* per year are calculated as:

$\boldsymbol{d}_{\boldsymbol{l}}\boldsymbol{=}\left( \frac{\boldsymbol{D}_{\boldsymbol{l}}}{\boldsymbol{dh}}\boldsymbol{*}\boldsymbol{n}_{\boldsymbol{l}}\boldsymbol{+}\boldsymbol{s}_{\boldsymbol{l}}\boldsymbol{*}\boldsymbol{n}_{\boldsymbol{l}}\boldsymbol{*}\boldsymbol{t}_{\boldsymbol{l}} \right)\boldsymbol{/8}$, where **(9.1.1)**

*D_l_* is average distance travelled per round of delivery at level *l*

*dh* is average speed per hour

*s_l_* is number of stores at level *l*

*n_l_* is number of deliveries at level *l*

*t_l_* is time in hours spent loading/unloading supplies per store

Scaling factor (*Z_l_*) captures the share of vehicle carriage capacity taken up by the vaccine and is computed as:

$\boldsymbol{Z}_{\boldsymbol{l}}\boldsymbol{=}\left( \frac{\boldsymbol{v}_{\boldsymbol{l}}\boldsymbol{+}\boldsymbol{vs}_{\boldsymbol{l}}}{\boldsymbol{s}_{\boldsymbol{l}}\boldsymbol{*}\boldsymbol{n}_{\boldsymbol{l}}}\boldsymbol{*m} \right)/{\boldsymbol{vv}_{\boldsymbol{l}}}$, where **(9.1.2)**

*v_l_* is volume of vaccines stored at level *l* in m3

*vs_l_* is volume of immunization supplies stored at level *l* in m3

*s_l_* is number of stores at level *l*

*n_l_* is number of deliveries at level *l*

*m* is number of stores per round of delivery at level l

*vv_l_* is vehicle carriage capacity at level l

Vehicle costs (*V*) and cost of maintenance (*M*) are estimated by the formula outlined above.

Cost of fuel (*F*) is calculated as per formula:

$\boldsymbol{F=}\sum_{\boldsymbol{l=1}}^{\boldsymbol{m}} \boldsymbol{D}_{\boldsymbol{l}}\boldsymbol{*}\boldsymbol{C}_{\boldsymbol{l}}\boldsymbol{*f*Z}$, where **(9.2)**

*l* is level of storage facility (i.e. central, regional, district, etc.)

*D_l_* is average distance travelled per round of delivery at level *l*

*C_l_* is fuel consumption of vehicle operating at store level *l*

*f* is cost of fuel per litre

*Z* is scaling factor

Cost of cold boxes is added to regional and district distribution stores and is calculated including 20% replacement rate as follows:

$\boldsymbol{S=}\sum_{\boldsymbol{l=1}}^{\boldsymbol{m}} \frac{\boldsymbol{v}_{\boldsymbol{l}}}{\boldsymbol{vb}_{\boldsymbol{l}}\boldsymbol{*}\boldsymbol{s}_{\boldsymbol{l}}}\boldsymbol{*}\boldsymbol{f}_{\boldsymbol{l}}\boldsymbol{*1.2*}\frac{\boldsymbol{v}}{\boldsymbol{V}}$, where **(9.3)**

*l* is level of storage facility (i.e. central, regional, district, etc.)

*v_l_* is volume of vaccines stored at level *l* in m3

*vb_l_* is internal volume of cold boxes used at level *l* in m3

*s_l_* is number of stores at level *l*

*f_l_* is cost of cold box used at level *l*

*v* is volume of RTS,S vaccine

*V* is total volume of all vaccines in EPI schedule

**Vaccination (*I*)**

Cost of an immunization is captured as a following formula:

$\boldsymbol{I=W+F+S}$, where **(10)**

W is cost of wages of medical stuff involved in immunization including outreach

F is cost of facility including overheads and furniture

S is cost of office supplies

Wages of immunization workers including fixed and outreach delivery are calculated as:

$\boldsymbol{W=}\left[ \frac{\boldsymbol{W}}{\boldsymbol{20*h*60}}\boldsymbol{*}\boldsymbol{t}_{\boldsymbol{d}}\boldsymbol{*B*C+}\boldsymbol{q}_{\boldsymbol{4}}\boldsymbol{*}\boldsymbol{d}_{\boldsymbol{4}}\boldsymbol{*12*}\frac{\boldsymbol{n}}{\boldsymbol{N}} \right]\boldsymbol{*}\boldsymbol{s}_{\boldsymbol{4}}$, where **(10.1)**

*W* is monthly wages of immunization staff

*h* is number of hours worked per day

*t_d_* if time to administer a dose in minutes

*B* is cohort size

*C* is proportion of birth cohort immunized; if multiple doses are required C is the sum of expected coverage for each dose

*q_4_* is immunization staff per-diem

*d_4_* is number of days per month staff conducts outreach

*n* is number of doses in RTS,S schedule

*N* is number of vaccine doses in EPI schedule

*s_4_* is number of health facilities providing immunization

Facility costs including overheads(10%) are estimated as follows:

$\boldsymbol{F=}\left( \boldsymbol{f}_{\boldsymbol{f}}\boldsymbol{*}\boldsymbol{s}_{\boldsymbol{f}}\boldsymbol{*}\boldsymbol{p}_{\boldsymbol{epi}}\boldsymbol{*}\frac{\boldsymbol{n}}{\boldsymbol{N}} \right)\boldsymbol{*1.1*}\boldsymbol{s}_{\boldsymbol{4}}$, where **(10.2)**

*f_f_* is annualized and discounted cost of facility per m2

*s_f_* is square footage of an immunization office

*p_epi_* is facility usage for EPI related activities

*n* is number of doses in RTS,S schedule

*N* is number of vaccine doses in EPI schedule

*s_4_* is number of health facilities providing immunization

Cost of supplies and office furnishings (S) are computed as:

$\boldsymbol{S=}\left[ \sum_{\boldsymbol{f=1}}^{\boldsymbol{n}} \boldsymbol{U}_{\boldsymbol{f}}\boldsymbol{*}\boldsymbol{n}_{\boldsymbol{f}}\boldsymbol{+}\sum_{\boldsymbol{s=1}}^{\boldsymbol{m}} \boldsymbol{U}_{\boldsymbol{s}}\boldsymbol{*}\boldsymbol{n}_{\boldsymbol{s}} \right]\boldsymbol{*}\boldsymbol{p}_{\boldsymbol{epi}}\boldsymbol{*}\frac{\boldsymbol{n}}{\boldsymbol{N}}\boldsymbol{*}\boldsymbol{s}_{\boldsymbol{4}}$, where **(10.3)**

*f* is unit of furniture (i.e. stool, chair, etc.)

*U_f_* is annualized and discounted cost per unit of furniture f

*n_f_* is number of units of furniture f per immunization office

*s* is unit of stationaries (i.e. pens, paper etc.)

*n_s_* is number of units of stationary s per immunization office

*p_epi_* is facility usage for EPI related activities

*n* is number of doses in RTS,S schedule

*N* is number of vaccine doses in EPI schedule

*s_4_* is number of health facilities providing immunization

**Waste management (*G*)**

Cost of waste management are summarized by the formula:

$\boldsymbol{G=W+E+F}$, where **(11)**

W is wages of technical staff operating the incinerator

E is cost of equipment including protective gear and maintenance and overheads

F is cost fuel

Wages of technical staff involved in waste management of immunization supplies are first attributed to EPI and then to RTS,S based on the total value of RTS,S in new immunization schedule and are calculated as follows:

$\boldsymbol{W=}\left[ \sum_{\boldsymbol{l=1}}^{\boldsymbol{e}} \boldsymbol{W}_{\boldsymbol{l}}\boldsymbol{*12*}\boldsymbol{s}_{\boldsymbol{l}}\boldsymbol{*}\boldsymbol{p}_{\boldsymbol{l}} \right]\boldsymbol{*}\frac{\boldsymbol{v}}{\boldsymbol{V}}\boldsymbol{*}\frac{\boldsymbol{v}_{\boldsymbol{i}}}{\boldsymbol{v}}$**,** where **(11.1)**

*l* is level of EPI facility

*W_l_* is monthly wages of technical staff at facility level l

*s_l_* is number of facilities at level l

*p_l_* is percent of time spent of EPI activities at level l

*v* is volume of RTS,S vaccine

*V* is total volume of all vaccines in EPI schedule

*v_i_* is volume of RTS,S incinerated

*In this paper in the absence of detailed information we assumed that incinerators are used at central, regional, and district level facilities; at all other levels immunization supplies are discharged at fire pits.*

Cost of incinerator and related equipment (*E*) is discounted and annualized and captured by the following formula:

$\boldsymbol{E=}\left[ \sum_{\boldsymbol{l=1}}^{\boldsymbol{e}} \sum_{\boldsymbol{c=1}}^{\boldsymbol{n}} \boldsymbol{(U}_{\boldsymbol{c}}\boldsymbol{+M+F)*}\boldsymbol{s}_{\boldsymbol{l}}\boldsymbol{*}\boldsymbol{p}_{\boldsymbol{l}} \right]\boldsymbol{*}\frac{\boldsymbol{v}}{\boldsymbol{V}}\boldsymbol{*}\frac{\boldsymbol{v}_{\boldsymbol{i}}}{\boldsymbol{v}}$**,** where **(11.2)**

*l* is level of EPI facility with an incinerator

*c* is equipment (i.e. incinerator, bottle crusher, protective gear)

*U_c_* is annualized and discounted cost of equipment *c*

*M* is equipment maintenance

*F* is equipment overheads

*s_l_* is number of facilities at level l

*p_l_* is percent of time spent of EPI activities at level l

*v* is volume of RTS,S vaccine

*V* is total volume of all vaccines in EPI schedule

*v_i_* is volume of RTS,S incinerated

*In this paper we cost an incinerator, bottle crasher, and protective gear for each facility equipped with an incinerator. We allocate 5% of equipment cost toward maintenance(M).*

Cost of fuel (*F*) is calculated as follows:

$\boldsymbol{F=f*Cf*12*}\left( \boldsymbol{s}\boldsymbol{4-s}\boldsymbol{1-s}\boldsymbol{2-s}\boldsymbol{3} \right)\boldsymbol{*}\boldsymbol{p}_{\boldsymbol{4}}\boldsymbol{*}\frac{\boldsymbol{v}}{\boldsymbol{V}}$, where **(11.4)**

*f* is cost of fuel per litre

*C_f_* is fuel consumption per incinerator per month

*s_1_-s_4_* is number of health facilities at each level

*p_4_* is proportion fire pit is used by EPI

*V* is total volume of all vaccines in EPI schedule

*v_i_* is volume of RTS,S incinerated

*In this paper we assume fuel consumption per month of 10l per facility (Cf=10). Proportion of time fire pit is used for EPI (p4) at lower levels is imputed based on time allocation for technical staff.*

**Supplementary Table S1: Scenarios for immunization delivery by schedule**

|  | **6-12 weeks** | **6-12 weeks 4 dose** | **6-9 months** | **6-9 months 4 dose** |
| --- | --- | --- | --- | --- |
| **Introduction** | Baseline | Baseline | Baseline | Baseline |
| **Supervision** | Baseline | Increase supervision by 5% | Increase supervision by 5% | Increase supervision by 10% |
| **Monitoring** | Baseline | Baseline | Baseline | Baseline |
| **Training** | Baseline | Baseline | Baseline | Baseline |
| **EIC** | Baseline | Increase advertisement by 10% of start-up advertisement budget annually (RTS,S specific messaging) | Increase advertisement by 10% of start-up advertisement budget annually (RTS,S specific messaging) | Increase advertisement by 10% of start-up advertisement budget annually (RTS,S specific messaging) and community outreach |
| **Procurement** | 3 doses, DTP coverage rate | 4 doses, DTP coverage rate for doses 1-3, 80% of 3^rd^ dose for 4^th^ dose | 3 doses, 75% of DTP coverage rate | 4 doses, 75% of DTP coverage rate for doses 1-3, 80% of 3^rd^ dose for 4^th^ dose |
| **Storage** | Baseline | Baseline | Baseline | Baseline |
| **Transportation** | Baseline | Baseline | Baseline | Baseline |
| **Vaccination** | Baseline | Increase time for immunization for 4^th^ dose to 7 minutes | Increase time for immunization for 2^nd^ dose to 7 minutes | Increase time for immunization for 2^nd^ and 4^th^ dose to 7 minutes |
| **Waste Management** | Baseline | Baseline | Baseline | Baseline |

**Supplementary Table S2: Average annual *economic* cost of RTS,S immunization delivered in the 6 to 9 months schedule by country (USD, 2013)**

|  | Burkina Faso | Ghana | Kenya | Senegal | Tanzania | Uganda |
| --- | --- | --- | --- | --- | --- | --- |
| Introduction^1^ | $167'494 | $301'400 | $513'607 | $183'233 | $410'628 | $260'915 |
| Supervision | $29'941 | $178'654 | $147'950 | $213'450 | $650'896 | $251'771 |
| Monitoring | $98'428 | $158'016 | $470'528 | $197'951 | $227'203 | $66'969 |
| Training^2^ | $3'084 | $7'415 | $17'447 | $4'892 | $12'256 | $9'630 |
| EIC^3^ | $94'352 | $97'099 | $96'976 | $47'450 | $109'641 | $60'151 |
| Procurement | $9'903'660 | $10'679'279 | $14'591'719 | $6'447'159 | $24'677'812 | $18'322'750 |
| Storage | $225'522 | $280'159 | $529'596 | $246'328 | $365'121 | $532'201 |
| Transportation | $74'320 | $124'292 | $303'080 | $84'534 | $378'273 | $262'421 |
| Vaccination | $359'144 | $618'568 | $2'946'024 | $519'996 | $1'970'238 | $1'232'853 |
| Waste Management | $14'711 | $25'040 | $57'725 | $27'364 | $44'795 | $37'857 |
| Total | $10'970'655 | $12'469'923 | $19'674'652 | $7'972'356 | $28'846'864 | $21'037'518 |
| Total Delivery^4^ | $1'066'995 | $1'790'644 | $5'082'933 | $1'525'197 | $4'169'051 | $2'714'768 |
| Total Per Dose | $7.45 | $7.85 | $9.07 | $8.32 | $7.86 | $7.72 |
| Total Delivery Per Dose | $0.73 | $1.13 | $2.34 | $1.59 | $1.14 | $1.00 |
| Total Per FIC^5^ | $23.11 | $24.08 | $28.28 | $25.49 | $24.62 | $24.80 |

**Notes:** ^1^ Annualized and discounted economic cost of activities held in the introductory stage (micro-planning, cold chain evaluation, training, etc.). See paper Table1 for details. ^2^ RTS,S fraction of costs for routine training activities conducted by the EPI program (assuming 10% annual turn-over); excluding training in advance of the new vaccine introduction costed under “Introduction”. ^3^ RTS,S fraction of costs for routine EIC activities conducted by the EPI program; excluding expenditures related to initial information and incentivisation of the population training in advance of the new vaccine introduction costed under “Introduction”. ^4^ Program costs net of vaccine and immunization supplies. ^5^ FIC is defined as a child that received 3 doses of the schedule.

**Supplementary Table S3: Average annual *financial* cost of RTS,S immunization delivered in the 6 to 9 months schedule by country (USD, 2013)**

|  | Burkina Faso | Ghana | Kenya | Senegal | Tanzania | Uganda |
| --- | --- | --- | --- | --- | --- | --- |
| Introduction^1^ | $149'812 | $264'083 | $386'215 | $144'032 | $334'038 | $221'328 |
| Supervision | $1'435 | $8'584 | $7'106 | $10'260 | $31'206 | $12'163 |
| Monitoring | $8'000 | $8'000 | $8'000 | $8'000 | $8'000 | $8'000 |
| Training^2^ | $0 | $0 | $0 | $0 | $0 | $0 |
| EIC^3^ | $39'918 | $44'136 | $43'101 | $21'089 | $47'580 | $24'768 |
| Procurement | $9'903'660 | $10'679'279 | $14'591'719 | $6'447'159 | $24'677'812 | $18'322'750 |
| Storage | $204'061 | $244'579 | $414'641 | $175'518 | $262'571 | $506'407 |
| Transportation | $39'225 | $30'357 | $79'547 | $30'170 | $142'293 | $102'807 |
| Vaccination | $80'235 | $103'743 | $364'297 | $17'345 | $185'542 | $270'581 |
| Waste Management | $9'925 | $8'339 | $39'792 | $7'819 | $29'449 | $30'681 |
| Total | $10'436'271 | $11'391'101 | $15'934'417 | $6'861'391 | $25'718'490 | $19'499'486 |
| Total Delivery^4^ | $532'611 | $711'822 | $1'342'698 | $414'233 | $1'040'679 | $1'176'735 |
| Total Per Dose | $7.09 | $7.17 | $7.34 | $7.16 | $7.01 | $7.16 |
| Total Delivery Per Dose | $0.36 | $0.45 | $0.62 | $0.43 | $0.28 | $0.43 |
| Total Per FIC^5^ | $21.99 | $22.00 | $22.90 | $21.94 | $21.95 | $22.99 |

**Notes:** ^1^ Annualized financial cost of activities held in the introductory stage (micro-planning, cold chain evaluation, training, etc.). See paper Table1 for details. ^2^ RTS,S fraction of costs for routine training activities conducted by the EPI program (assuming 10% annual turn-over); excluding training in advance of the new vaccine introduction costed under “Introduction”. ^3^ RTS,S fraction of costs for routine EIC activities conducted by the EPI program; excluding expenditures related to initial information and incentivisation of the population training in advance of the new vaccine introduction costed under “Introduction”. ^4^ Program costs net of vaccine and immunization supplies. ^5^ FIC is defined as a child that received 3 doses of the schedule.

**Supplementary Table S4: Average annual *economic* cost of RTS,S immunization delivered in the 6-9 months 4 dose schedule by country (USD, 2013)**

|  | Burkina Faso | Ghana | Kenya | Senegal | Tanzania | Uganda |
| --- | --- | --- | --- | --- | --- | --- |
| Introduction^1^ | $167'494 | $301'400 | $513'607 | $183'233 | $410'628 | $260'915 |
| Supervision | $40'005 | $239'950 | $198'394 | $286'226 | $871'306 | $335'695 |
| Monitoring | $123'329 | $200'329 | $600'036 | $251'138 | $288'093 | $83'051 |
| Training^2^ | $3'933 | $9'506 | $22'332 | $6'262 | $15'661 | $12'256 |
| EIC^3^ | $119'247 | $148'345 | $177'567 | $74'682 | $172'492 | $87'738 |
| Procurement | $12'457'572 | $13'465'178 | $18'335'080 | $8'129'821 | $30'981'478 | $22'886'980 |
| Storage | $278'096 | $344'984 | $646'740 | $301'969 | $446'716 | $644'744 |
| Transportation | $94'405 | $157'207 | $378'992 | $106'725 | $475'822 | $325'050 |
| Vaccination | $463'789 | $806'249 | $3'820'746 | $683'676 | $2'563'441 | $1'585'290 |
| Waste Management | $18'122 | $30'803 | $70'432 | $33'521 | $54'695 | $45'825 |
| Total | $13'765'992 | $15'703'951 | $24'763'924 | $10'057'252 | $36'280'332 | $26'267'544 |
| Total Delivery^4^ | $1'308'420 | $2'238'773 | $6'428'845 | $1'927'431 | $5'298'855 | $3'380'564 |
| Total Per Dose | $7.43 | $7.84 | $9.08 | $8.32 | $7.88 | $7.72 |
| Total Delivery Per Dose | $0.71 | $1.12 | $2.36 | $1.59 | $1.15 | $0.99 |
| Total Per FIC^5^ | $36.25 | $37.91 | $44.49 | $40.20 | $38.71 | $38.71 |

**Notes:** ^1^ Annualized and discounted economic cost of activities held in the introductory stage (micro-planning, cold chain evaluation, training, etc.). See paper Table1 for details. ^2^ RTS,S fraction of costs for routine training activities conducted by the EPI program (assuming 10% annual turn-over); excluding training in advance of the new vaccine introduction costed under “Introduction”. ^3^ RTS,S fraction of costs for routine EIC activities conducted by the EPI program; excluding expenditures related to initial information and incentivisation of the population training in advance of the new vaccine introduction costed under “Introduction”. ^4^ Program costs net of vaccine and immunization supplies. ^5^ FIC is defined as a child that received 4 doses of the schedule.

**Supplementary Table S5: Average annual *financial* cost of RTS,S immunization delivered in the 6-9 months 4 dose schedule by country (USD, 2013)**

|  | Burkina Faso | Ghana | Kenya | Senegal | Tanzania | Uganda |
| --- | --- | --- | --- | --- | --- | --- |
| Introduction^1^ | $149'812 | $264'083 | $386'215 | $144'032 | $334'038 | $221'328 |
| Supervision | $3'659 | $22'011 | $18'191 | $26'267 | $79'748 | $30'961 |
| Monitoring | $8'000 | $8'000 | $8'000 | $8'000 | $8'000 | $8'000 |
| Training^2^ | $0 | $0 | $0 | $0 | $0 | $0 |
| EIC^3^ | $48'942 | $75'197 | $94'872 | $36'109 | $86'073 | $41'699 |
| Procurement | $12'457'572 | $13'465'178 | $18'335'080 | $8'129'821 | $30'981'478 | $22'886'980 |
| Storage | $251'392 | $300'876 | $505'929 | $215'013 | $320'624 | $612'997 |
| Transportation | $50'734 | $40'028 | $102'725 | $38'906 | $183'101 | $129'179 |
| Vaccination | $102'329 | $133'004 | $466'300 | $22'201 | $237'081 | $344'375 |
| Waste Management | $12'226 | $10'258 | $48'552 | $9'578 | $35'958 | $37'138 |
| Total | $13'084'667 | $14'318'635 | $19'965'864 | $8'629'928 | $32'266'102 | $24'312'656 |
| Total Delivery^4^ | $627'095 | $853'457 | $1'630'783 | $500'107 | $1'284'624 | $1'425'676 |
| Total Per Dose | $7.06 | $7.15 | $7.32 | $7.14 | $7.00 | $7.14 |
| Total Delivery Per Dose | $0.34 | $0.43 | $0.60 | $0.41 | $0.28 | $0.42 |
| Total Per FIC^5^ | $34.46 | $34.57 | $35.87 | $34.49 | $34.42 | $35.82 |

**Notes:** ^1^ Annualized financial cost of activities held in the introductory stage (micro-planning, cold chain evaluation, training, etc.). See paper Table1 for details. ^2^ RTS,S fraction of costs for routine training activities conducted by the EPI program (assuming 10% annual turn-over); excluding training in advance of the new vaccine introduction costed under “Introduction”. ^3^ RTS,S fraction of costs for routine EIC activities conducted by the EPI program; excluding expenditures related to initial information and incentivisation of the population training in advance of the new vaccine introduction costed under “Introduction”. ^4^ Program costs net of vaccine and immunization supplies. ^5^ FIC is defined as a child that received 4 doses of the schedule.

**Supplementary Table S6: Average annual *economic* cost of RTS,S immunization delivered in the 6-12 weeks schedule by country (USD, 2013)**

|  | Burkina Faso | Ghana | Kenya | Senegal | Tanzania | Uganda |
| --- | --- | --- | --- | --- | --- | --- |
| Introduction^1^ | $167'494 | $301'400 | $513'607 | $183'233 | $410'628 | $260'915 |
| Supervision | $28'516 | $170'146 | $140'905 | $203'286 | $619'901 | $239'782 |
| Monitoring | $98'428 | $158'016 | $470'528 | $197'951 | $227'203 | $66'969 |
| Training^2^ | $3'084 | $7'415 | $17'447 | $4'892 | $12'256 | $9'630 |
| EIC^3^ | $54'434 | $52'963 | $53'876 | $26'361 | $62'061 | $35'383 |
| Procurement | $13'204'882 | $14'239'040 | $19'455'626 | $8'596'211 | $32'903'752 | $24'430'332 |
| Storage | $293'093 | $362'459 | $680'541 | $316'849 | $470'787 | $681'289 |
| Transportation | $100'347 | $166'382 | $401'592 | $112'884 | $505'633 | $346'021 |
| Vaccination | $382'637 | $676'571 | $3'184'340 | $595'248 | $2'184'796 | $1'314'586 |
| Waste Management | $19'094 | $32'354 | $74'094 | $35'166 | $57'607 | $48'409 |
| Total | $14'352'008 | $16'166'747 | $24'992'554 | $10'272'081 | $37'454'624 | $27'433'314 |
| Total Delivery^4^ | $1'147'126 | $1'927'707 | $5'536'929 | $1'675'870 | $4'550'874 | $3'002'983 |
| Total Per Dose | $7.31 | $7.64 | $8.64 | $8.04 | $7.66 | $7.55 |
| Total Delivery Per Dose | $0.58 | $0.91 | $1.91 | $1.31 | $0.93 | $0.83 |
| Total Per FIC^5^ | $22.68 | $23.42 | $26.94 | $24.63 | $23.98 | $24.25 |

**Notes:** ^1^ Annualized and discounted economic cost of activities held in the introductory stage (micro-planning, cold chain evaluation, training, etc.). See paper Table1 for details. ^2^ RTS,S fraction of costs for routine training activities conducted by the EPI program (assuming 10% annual turn-over); excluding training in advance of the new vaccine introduction costed under “Introduction”. ^3^ RTS,S fraction of costs for routine EIC activities conducted by the EPI program; excluding expenditures related to initial information and incentivisation of the population training in advance of the new vaccine introduction costed under “Introduction”. ^4^ Program costs net of vaccine and immunization supplies. ^5^ FIC is defined as a child that received 3 doses of the schedule.

**Supplementary Table S7: Average annual *financial* cost of RTS,S immunization delivered in the 6-12 weeks schedule by country (USD, 2013)**

|  | Burkina Faso | Ghana | Kenya | Senegal | Tanzania | Uganda |
| --- | --- | --- | --- | --- | --- | --- |
| Introduction^1^ | $149'812 | $264'083 | $386'215 | $144'032 | $334'038 | $221'328 |
| Supervision | $0 | $0 | $0 | $0 | $0 | $0 |
| Monitoring | $8'000 | $8'000 | $8'000 | $8'000 | $8'000 | $8'000 |
| Training^2^ | $0 | $0 | $0 | $0 | $0 | $0 |
| EIC^3^ | $0 | $0 | $0 | $0 | $0 | $0 |
| Procurement | $13'204'882 | $14'239'040 | $19'455'626 | $8'596'211 | $32'903'752 | $24'430'332 |
| Storage | $264'874 | $316'031 | $532'236 | $225'565 | $337'701 | $647'563 |
| Transportation | $54'199 | $42'827 | $109'851 | $41'382 | $195'855 | $138'154 |
| Vaccination | $80'235 | $103'743 | $364'297 | $17'345 | $185'542 | $270'581 |
| Waste Management | $12'882 | $10'774 | $51'076 | $10'048 | $37'872 | $39'232 |
| Total | $13'774'884 | $14'984'499 | $20'907'300 | $9'042'583 | $34'002'760 | $25'755'190 |
| Total Delivery^4^ | $570'002 | $745'459 | $1'451'675 | $446'372 | $1'099'008 | $1'324'857 |
| Total Per Dose | $7.02 | $7.08 | $7.23 | $7.08 | $6.95 | $7.09 |
| Total Delivery Per Dose | $0.29 | $0.35 | $0.50 | $0.35 | $0.23 | $0.37 |
| Total Per FIC^5^ | $21.76 | $21.70 | $22.54 | $21.69 | $21.77 | $22.77 |

**Notes:** ^1^ Annualized financial cost of activities held in the introductory stage (micro-planning, cold chain evaluation, training, etc.). See paper Table1 for details. ^2^ RTS,S fraction of costs for routine training activities conducted by the EPI program (assuming 10% annual turn-over); excluding training in advance of the new vaccine introduction costed under “Introduction”. ^3^ RTS,S fraction of costs for routine EIC activities conducted by the EPI program; excluding expenditures related to initial information and incentivisation of the population training in advance of the new vaccine introduction costed under “Introduction”. ^4^ Program costs net of vaccine and immunization supplies. ^5^ FIC is defined as a child that received 3 doses of the schedule.

**Supplementary Table S8: Average annual *economic* cost of RTS,S immunization delivered in the 6-12 weeks 4 dose schedule by country (USD, 2013)**

|  | Burkina Faso | Ghana | Kenya | Senegal | Tanzania | Uganda |
| --- | --- | --- | --- | --- | --- | --- |
| Introduction^1^ | $167'494 | $301'400 | $513'607 | $183'233 | $410'628 | $260'915 |
| Supervision | $38'186 | $229'043 | $189'376 | $273'216 | $831'701 | $320'436 |
| Monitoring | $123'329 | $200'329 | $600'036 | $251'138 | $288'093 | $83'051 |
| Training^2^ | $3'933 | $9'506 | $22'332 | $6'262 | $15'661 | $12'256 |
| EIC^3^ | $109'341 | $112'038 | $112'062 | $54'831 | $126'880 | $69'801 |
| Procurement | $16'610'096 | $17'953'570 | $24'446'774 | $10'839'762 | $41'308'640 | $30'515'972 |
| Storage | $359'327 | $443'320 | $824'538 | $385'376 | $571'893 | $818'530 |
| Transportation | $127'765 | $210'599 | $501'610 | $142'557 | $636'308 | $427'772 |
| Vaccination | $501'418 | $899'559 | $4'201'717 | $804'756 | $2'905'881 | $1'714'530 |
| Waste Management | $23'379 | $39'519 | $89'667 | $42'730 | $69'792 | $58'096 |
| Total | $18'064'268 | $20'398'882 | $31'501'718 | $12'983'860 | $47'165'476 | $34'281'360 |
| Total Delivery^4^ | $1'454'171 | $2'445'312 | $7'054'944 | $2'144'099 | $5'856'837 | $3'765'388 |
| Total Per Dose | $7.31 | $7.64 | $8.67 | $8.06 | $7.68 | $7.56 |
| Total Delivery Per Dose | $0.59 | $0.92 | $1.94 | $1.33 | $0.95 | $0.83 |
| Total Per FIC^5^ | $35.68 | $36.93 | $42.45 | $38.92 | $37.74 | $37.89 |

**Notes:** ^1^ Annualized and discounted economic cost of activities held in the introductory stage (micro-planning, cold chain evaluation, training, etc.). See paper Table1 for details. ^2^ RTS,S fraction of costs for routine training activities conducted by the EPI program (assuming 10% annual turn-over); excluding training in advance of the new vaccine introduction costed under “Introduction”. ^3^ RTS,S fraction of costs for routine EIC activities conducted by the EPI program; excluding expenditures related to initial information and incentivisation of the population training in advance of the new vaccine introduction costed under “Introduction”. ^4^ Program costs net of vaccine and immunization supplies. ^5^ FIC is defined as a child that received 4 doses of the schedule.

**Supplementary Table S9: Average annual *financial* cost of RTS,S immunization delivered in the 6-12 weeks 4 dose schedule by country (USD, 2013)**

|  | Burkina Faso | Ghana | Kenya | Senegal | Tanzania | Uganda |
| --- | --- | --- | --- | --- | --- | --- |
| Introduction^1^ | $149'812 | $264'083 | $386'215 | $144'032 | $334'038 | $221'328 |
| Supervision | $1'830 | $11'005 | $9'095 | $13'133 | $39'874 | $15'480 |
| Monitoring | $8'000 | $8'000 | $8'000 | $8'000 | $8'000 | $8'000 |
| Training^2^ | $0 | $0 | $0 | $0 | $0 | $0 |
| EIC^3^ | $39'918 | $44'136 | $43'101 | $21'089 | $47'580 | $24'768 |
| Procurement | $16'610'096 | $17'953'570 | $24'446'774 | $10'839'762 | $41'308'640 | $30'515'972 |
| Storage | $324'319 | $386'030 | $644'126 | $274'093 | $409'165 | $777'167 |
| Transportation | $70'509 | $56'903 | $142'561 | $53'595 | $253'205 | $173'805 |
| Vaccination | $102'329 | $133'004 | $466'300 | $22'201 | $237'081 | $344'375 |
| Waste Management | $15'772 | $13'160 | $61'812 | $12'210 | $45'883 | $47'082 |
| Total | $17'322'584 | $18'869'892 | $26'207'984 | $11'388'116 | $42'683'468 | $32'127'978 |
| Total Delivery^4^ | $712'489 | $916'322 | $1'761'209 | $548'354 | $1'374'826 | $1'612'005 |
| Total Per Dose | $7.01 | $7.07 | $7.21 | $7.07 | $6.95 | $7.08 |
| Total Delivery Per Dose | $0.29 | $0.34 | $0.49 | $0.34 | $0.22 | $0.36 |
| Total Per FIC^5^ | $34.21 | $34.17 | $35.31 | $34.14 | $34.15 | $35.51 |

**Notes:** ^1^ Annualized financial cost of activities held in the introductory stage (micro-planning, cold chain evaluation, training, etc.). See paper Table1 for details. ^2^ RTS,S fraction of costs for routine training activities conducted by the EPI program (assuming 10% annual turn-over); excluding training in advance of the new vaccine introduction costed under “Introduction”. ^3^ RTS,S fraction of costs for routine EIC activities conducted by the EPI program; excluding expenditures related to initial information and incentivisation of the population training in advance of the new vaccine introduction costed under “Introduction”. ^4^ Program costs net of vaccine and immunization supplies. ^5^ FIC is defined as a child that received 4 doses of the schedule.

**Supplementary Table S10: Total *economic* cost of RTS,S related introductory stage activities by country (USD, 2013)**

|  | **Burkina Faso** | **Ghana** | **Kenya** | **Senegal** | **Tanzania** | **Uganda** |
| --- | --- | --- | --- | --- | --- | --- |
| Planning | $31'727 | $41'004 | $47'509 | $49'971 | $97'497 | $105'236 |
| Cold store assessment | $73'710 | $73'710 | $73'710 | $73'710 | $73'710 | $73'710 |
| Revision of immunization cards and tally sheets | $45'361 | $48'697 | $78'318 | $28'651 | $108'942 | $91'952 |
| Training of trainers | $85'146 | $390'909 | $400'632 | $186'201 | $311'715 | $157'598 |
| Training of supervisors | $41'871 | $25'772 | $43'565 | $49'847 | $78'752 | $168'918 |
| Training of vaccinators | $99'107 | $201'239 | $951'566 | $155'320 | $549'168 | $347'573 |
| Social Mobilization and IEC | $510'775 | $816'052 | $1'126'750 | $427'413 | $956'490 | $437'829 |
| Total | $887'697 | $1'597'382 | $2'722'050 | $971'112 | $2'176'275 | $1'382'816 |
| Total annualized and discounted^1^ | $167'494 | $301'400 | $513'607 | $183'233 | $410'628 | $260'915 |

**Notes:** ^1^ Annualized over 5 years and discounted at 3%

**Supplementary Table S11: Total *financial* cost of RTS,S related introductory stage activities by country (USD, 2013)**

|  | **Burkina Faso** | **Ghana** | **Kenya** | **Senegal** | **Tanzania** | **Uganda** |
| --- | --- | --- | --- | --- | --- | --- |
| Planning | $19'662 | $19'960 | $18'741 | $29'690 | $47'707 | $64'198 |
| Cold store assessment | $0 | $0 | $0 | $0 | $0 | $0 |
| Revision of immunization cards and tally sheets | $45'361 | $48'697 | $78'318 | $28'651 | $108'942 | $91'952 |
| Training of trainers | $76'742 | $352'221 | $346'384 | $162'885 | $245'561 | $123'221 |
| Training of supervisors | $37'503 | $24'079 | $36'985 | $43'869 | $64'371 | $147'107 |
| Training of vaccinators | $68'871 | $113'451 | $464'397 | $79'356 | $319'808 | $252'898 |
| Social Mobilization and IEC | $500'923 | $762'008 | $986'249 | $375'709 | $883'800 | $427'262 |
| Total | $749'061 | $1'320'416 | $1'931'073 | $720'160 | $1'670'190 | $1'106'638 |
| Total annualized^1^ | $149'812 | $264'083 | $386'215 | $144'032 | $334'038 | $221'328 |

**Notes:** ^1^ Annualized over 5 years

**Supplementary Table S12: Vaccine Volume Calculator**

|  |  |  |  |  |  |  |  | Volume (m3) | | | | | |
| --- | --- | --- | --- | --- | --- | --- | --- | --- | --- | --- | --- | --- | --- |
| Vaccine | Presentation | Vaccine vol. (cm3/dose) | Diluent vol. (cm3/dose) | Wastage rate | Age | Doses | Temperature | Burkina Faso | Ghana | Kenya | Senegal | Tanzania | Uganda |
| BCG | 20 | 1.3 | 1.1 | 0.5 | Birth | 1 | 2 to 8C | 3.19 | 3.76 | 5.88 | 2.49 | 9.18 | 7.26 |
| OPV | 20 | 2 |  | 0.25 | Birth, 4, 8, 12 weeks | 4 | -15 to -20C | 6.12 | 7.37 | 12.87 | 4.82 | 17.84 | 13.42 |
| DTP | 10 | 2.8 |  | 0.25 | Birth, 4, 8, 12 weeks | 3 | 2 to 8C | 6.57 | 7.82 | 13.02 | 5.35 | 19.56 | 14.35 |
| MCV | 10 | 2.6 | 3.2 | 0.4 | 9 months | 2 | 2 to 8C | 10.23 | 13.06 | 26.45 | 8.25 | 35.18 | 24.32 |
| PCV | 1 | 12 |  | 0.05 | 12-15 months | 3 | 2 to 8C | 20.04 | 23.56 | 40.89 | 13.86 | 60.60 | 41.85 |
| Rota | 1 | 17.1 |  | 0.05 | 6 and 10 weeks | 3 | 2 to 8C | 31.70 | 24.45 | 41.84 | 17.19 | 59.55 | 0.00 |
| HPV | 2 | 5.7 |  | 0.05 | 9 to 13 years |  |  |  | 44.68 | 72.31 | 29.55 | 106.35 | 74.92 |
| YFV | 10 | 2.6 | 4.9 | 0.4 | 9 months | 1 | 3 to 8C | 7.01 | 8.25 | 0.37 | 5.27 |  |  |
| TT | 10 | 3.1 |  | 0.25 | Female 15-49 | 2 | 2 to 8C | 23.01 | 58.58 | 48.63 | 25.02 | 109.68 | 84.18 |
|  |  |  |  |  |  |  |  |  |  |  |  |  |  |
| RTS,S EPI | 2 | 4.84 | 4.84 | 0.25 | 4, 8, 12 weeks | 3 | 2 to 8C | 22.73 | 27.04 | 45.00 | 18.49 | 67.61 | 49.60 |
| RTS,S EPI booster | 2 | 4.84 | 4.84 | 0.25 | 4, 8, 12 weeks, 18 months | 4 | 2 to 8C | 28.59 | 34.09 | 56.54 | 23.32 | 84.88 | 61.95 |
| RTS,S 5 to 17 months | 2 | 4.84 | 4.84 | 0.25 | 6, 7.5,9 months | 3 | 2 to 8C | 17.05 | 20.28 | 33.75 | 13.87 | 50.71 | 37.20 |
| RTS,S 5 to 17 months booster | 2 | 4.84 | 4.84 | 0.25 | 6, 7.5,9, 17 months | 4 | 2 to 8C | 21.44 | 25.57 | 42.40 | 17.49 | 63.66 | 46.46 |
| Net volume -20C |  |  |  |  |  |  |  | 6.12 | 7.37 | 13.02 | 5.35 | 19.56 | 14.35 |
| Net volume at +5 C |  |  |  |  |  |  |  | 101.76 | 184.17 | 243.35 | 103.96 | 389.19 | 238.70 |
| **RTS,S percent increase in volume** | | | | | | | | | | | | | |
| **EPI** |  |  |  |  |  |  |  | **17.4%** | 12.4% | **14.93%** | **14.47%** | **14.19%** | **16.39%** |
| **EPI booster** |  |  |  |  |  |  |  | **20.9%** | 15.1% | **18.07%** | **17.58%** | **17.19%** | **19.67%** |
| **5 to 17 months** |  |  |  |  |  |  |  | **13.6%** | 9.6% | **11.63%** | **11.26%** | **11.04%** | **12.82%** |
| **5 to 17 months booster** | |  |  |  |  |  |  | **16.6%** | 11.8% | **14.19%** | **13.79%** | **13.48%** | **15.51%** |

**Source:** <http://data.unicef.org/child-health/immunization> (WUENIC, 2013 revision) (2013) **;**

**Notes:** EPI schedule updated with planned vaccine introduction prior to RTS,S (tentatively in 2017); these include HPV, Rota, PCV, MCV2. HPV coverage is assumed to be 75% of DTP.
